# B cell-derived nociceptin/orphanin FQ contributes to impaired glucose tolerance and insulin resistance in obesity

**DOI:** 10.1016/j.isci.2025.112819

**Published:** 2025-06-04

**Authors:** Stephanie C. Puente-Ruiz, Leona Ide, Julia Schuller, Adel Ben-Kraiem, Anne Hoffmann, Adhideb Ghosh, Falko Noé, Christian Wolfrum, Kerstin Krause, Martin Gericke, Nora Klöting, Jens C. Brüning, F. Thomas Wunderlich, Matthias Blüher, Alexander Jais

**Affiliations:** 1 Helmholtz Institute for Metabolic, Obesity and Vascular Research (HI-MAG) of the Helmholtz Zentrum München at the University of Leipzig and University Hospital Leipzig, Leipzig, Germany; 2 Institute of Food, Nutrition and Health, ETH Zurich, Schwerzenbach, Switzerland; 3 Medical Department III - Endocrinology, Nephrology, Rheumatology, University of Leipzig Medical Center, Leipzig, Germany; 4 Institute of Anatomy, Leipzig University, Leipzig, Germany; 5 Max Planck Institute for Metabolism Research, Cologne, Germany

**Keywords:** Biological sciences, Endocrinology, Natural sciences, Physiology

## Abstract

Immune-derived opioid peptides have been implicated in immune regulation and inflammatory processes. Here, we investigate the effects of nociceptin/orphanin FQ (N/OFQ) on metabolic function and inflammation in obesity. Selectively targeting N/OFQ, encoded by the *Pnoc* gene, in B cells mitigates the adverse metabolic effects of diet-induced obesity and enhances insulin sensitivity and glucose tolerance. Notably, B cell-specific *Pnoc* knockout mice display a marked reduction in markers of immune cell migration and diminished macrophage recruitment in adipose tissue and liver. Mechanistically, we identify that N/OFQ promotes macrophage recruitment and metabolic inflammation, exacerbating glucose intolerance and insulin resistance during obesity. Overall, the immunomodulatory properties exhibited by the N/OFQ-NOP system render it a promising therapeutic target for mitigating metabolic inflammation.

## Highlights

• B cell-derived N/OFQ impairs glucose metabolism and promotes insulin resistance in obesity
• N/OFQ-NOP signaling enhances macrophage recruitment and drives metabolic inflammation
• *Pnoc* knockout in B cells reduces immune cell infiltration in adipose tissue and liver
• Targeting B cell-derived N/OFQ improves insulin sensitivity without affecting body weight

## Graphical abstract

**Figure:**
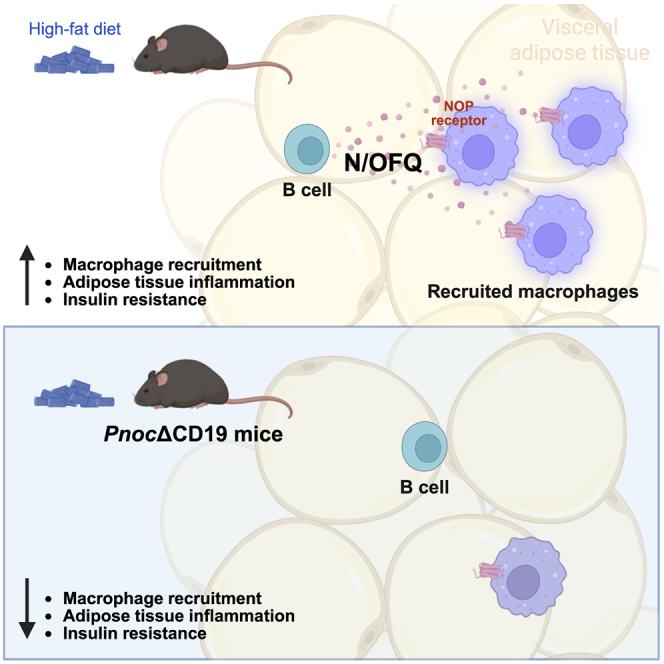

## Introduction

Obesity is a persistent metabolic disorder characterized by chronic low-grade inflammation, with immune cells playing a major role in disease progression.^1^^,^^2^^,^^3^ Among the various molecular mediators involved in immune regulation, endogenous opioid peptides, primarily known for their roles in nociception and stress responses, modulate immune function through their influence on cytokine production and immune cell migration.^4^^,^^5^^,^^6^ The genes encoding these peptides share a common ancestral origin and diversified through gene duplication events during early vertebrate evolution.^7^ The opioid/orphanin gene family comprises four opioid peptide precursors: proopiomelanocortin (POMC), prodynorphin (PDYN), proenkephalin (PENK), and prepronociceptin (PNOC). Notably, PNOC, identified in 1995, is the most recently discovered member of the opioid/orphanin gene family.^8^^,^^9^ The *PNOC* gene encodes the prepronociceptin precursor protein. This precursor is cleaved into the active peptides nociceptin/orphanin FQ (N/OFQ), a 17-amino acid peptide, and nocistatin.^10^^,^^11^ PNOC-expressing neurons in the central nervous system (CNS) modulate various physiological and behavioral functions.^12^^,^^13^^,^^14^^,^^15^^,^^16^ Beyond the CNS, N/OFQ also exerts effects on peripheral tissues, including the regulation of cardiovascular, gastrointestinal, urinary, and immune system functions.^17^^,^^18^^,^^19^ Upon release, N/OFQ engages its cognate nociceptin opioid peptide receptor (NOP, encoded by the *OPRL1* gene), which is widely distributed in peripheral tissues, including the gastrointestinal tract and immune cells.^20^^,^^21^^,^^22^ Notably, NOP receptor expression has been detected on circulating monocytes and lymphocytes.^23^^,^^24^^,^^25^^,^^26^ While the NOP receptor shares structural and functional features with classical μ-, δ-, and κ-opioid receptors, it is pharmacologically distinct, exhibiting low affinity for other opioid peptides and the opioid antagonist naloxone.^27^ Conversely, its endogenous agonist N/OFQ does not engage with classical opioid receptors.^28^

Recent studies suggest that N/OFQ plays a role in immune cell function and the regulation of inflammation.^26^^,^^29^^,^^30^^,^^31^^,^^32^^,^^33^ Preclinical studies using rodent models have shown that recombinant N/OFQ administration exacerbates inflammation and increases mortality in sepsis.^34^ Consistent with these findings, clinical studies have reported elevated serum N/OFQ levels in non-surviving septic patients.^35^ The absence of the NOP receptor in NOPR-deficient mice leads to reduced inflammatory cell infiltration and crypt distortion in a chemically induced colitis model compared to their wild-type littermates.^36^ Importantly, N/OFQ has been shown to stimulate monocyte chemotaxis, with this chemotactic response specifically mediated through the NOP receptor. Notably, naloxone does not significantly alter N/OFQ-induced monocyte chemotaxis, while UFP-101, a selective antagonist of the NOP receptor, effectively blocks this effect.^37^ Stimulation of T cells with lipopolysaccharide (LPS) and peptidoglycan G (PepG) upregulates NOP receptor expression, thereby enhancing N/OFQ binding and triggering changes in T cell migration and cytokine secretion.^38^ Additionally, N/OFQ recruits neutrophils to sites of inflammation.^30^ In arthritis, N/OFQ has been detected in synovial fluid, likely due to secretion by polymorphonuclear cells (PMNs). This neutrophil-derived N/OFQ may contribute to the development of neurogenic inflammation.^31^

Notably, a distinct PNOC isoform, characterized by a novel 5′ exon (ImEx2b) is expressed in immune cells. This exon, located upstream of the conventional exon 3, is transcribed from an alternative promoter. The immune-specific isoform is predominantly expressed in B cells and is upregulated following mitogen activation, suggesting a specialized role in modulating immune responses.^29^ B cells play a significant role in adipose tissue inflammation associated with obesity as well as aging.^39^^,^^40^^,^^41^^,^^42^ Studies have shown that during the development of obesity, the number of B cells increases within adipose tissue and their absence leads to improved glucose tolerance and insulin sensitivity.^42^^,^^43^ B cells from obese mice contribute to inflammation by producing pathogenic IgG antibodies and secreting a proinflammatory cytokine profile.^41^ B cells affect macrophage function through the secretion of chemokines that influence immune cell function and migration. These interactions between innate and adaptive immune cells, along with adipocytes, play a major role in the progression of obesity and type 2 diabetes. Given the evidence linking N/OFQ to immune regulation and inflammation, we hypothesize that B cell-derived N/OFQ contributes to immune cell function in adipose tissue and promotes metabolic inflammation in obesity.

## Results

### Correlation of *PNOC* expression with B cell markers in human visceral adipose tissue

**Figure 1:**
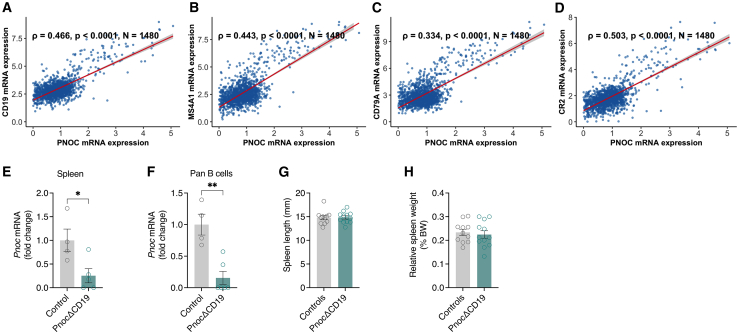
Correlation of *PNOC* expression with B cell markers in human visceral adipose tissue (A) Scatterplot showing the correlation between *CD19* and *PNOC* expression levels in human visceral adipose tissue samples (*n* = 1,480). Each point represents an individual sample, and the regression line indicates the relationship between the two genes (ρ = 0.466, *p* < 0.0001). (B) Scatterplot showing the correlation between *MS4A1* (CD20) and *PNOC* expression levels in human visceral adipose tissue samples (*n* = 1,480, ρ = 0.443, *p* < 0.0001). (C) Scatterplot showing the correlation between *CD79A* and *PNOC* expression levels in human visceral adipose tissue samples (*n* = 1,480, ρ = 0.334, *p* < 0.0001). (D) Scatterplot showing the correlation between *CR2* and *PNOC* expression levels in human visceral adipose tissue samples (*n* = 1,480, ρ = 0.503, *p* < 0.0001). (E) Quantification of *Pnoc* mRNA levels in spleens from *Pnoc*^wt/wt^::Cd19Cre^tg/wt^ control mice (*n* = 4) and *Pnoc*ΔCD19 mice (*Pnoc*^fl/fl^::Cd19Cre^tg/wt^, *n* = 4). (F) Quantification of *Pnoc* mRNA levels in isolated splenic B cells from control mice (*n* = 4) and *Pnoc*ΔCD19 mice (*n* = 4). (G) Spleen length from control mice (*n* = 11) and *Pnoc*ΔCD19 mice (*n* = 12). (H) Relative spleen weights in percent body weight from control mice (*n* = 11) and *Pnoc*ΔCD19 mice (*n* = 12). Gene correlations (Figures A–D) were assessed using Spearman’s rank correlation method. Scatterplots display the relationship between *PNOC* and individual gene expression levels, overlaid with a linear regression line and 95% confidence interval. Statistical analyses for (E)–(H) were performed using a two-tailed Student’s t test. Data are presented as mean ± SEM. ∗*p* < 0.05 and ∗∗*p* < 0.01.

To investigate the expression of *PNOC* mRNA in human adipose tissue, we conducted an analysis using an RNA sequencing dataset that includes 1,480 visceral adipose tissue samples obtained from the Leipzig Obesity BioBank (LOBB). *PNOC* mRNA expression was strongly correlated with multiple B cell markers (Figures 1A–1D). Specifically, *PNOC* correlated with the pan-B cell marker *CD19* (ρ = 0.466, *p* < 0.0001; Figure 1A), *MS4A1* (CD20, ρ = 0.443, *p* < 0.0001; Figure 1B), *CD79A* (ρ = 0.334, *p* < 0.0001; Figure 1C), and *CR2* (ρ = 0.503, *p* < 0.0001; Figure 1D). Conversely, *PNOC* mRNA levels were inversely correlated with pan macrophage markers (ρ = −0.301, *p* < 0.0001 for *CD68*, ρ = −0.388, *p* < 0.0001 for *CSF1R*; Figure S1A). *PNOC* expression also aligned positively with *NOS2*, a marker of pro-inflammatory M1 macrophages (ρ = 0.327, *p* < 0.0001), while showing an inverse relationship with *CD163*, indicative of anti-inflammatory M2 macrophages (ρ = −0.323, *p* < 0.0001; Figure S1). To assess the relevance of *PNOC* expression in relation to systemic metabolic parameters, we leveraged the adiposetissue.org platform.^44^ In this multi-cohort database, *PNOC* expression displayed a significant positive correlation with several clinical parameters linked to metabolic dysfunction, including HOMA-IR, waist-to-hip ratio (WHR), waist circumference, body mass index (BMI), as well as circulating levels of insulin, glucose, triglycerides, and low-density lipoprotein (LDL). Conversely, *PNOC* expression was inversely correlated with circulating high-density lipoprotein (HDL) levels (Figure S1C).

To investigate the functional role of *Pnoc* expression in B cells, we generated a mouse line with a conditional *Pnoc* allele by flanking exon 3 of the *Pnoc* gene with loxP sites (*Pnoc*^fl/fl^) and crossing these mice with Cd19-Cre transgenic animals^45^ (Figure S1D). The resulting mice (*Pnoc*^fl/fl^::*Cd19*Cre^tg/wt^), hereafter referred to as *Pnoc*ΔCD19, exhibit B cell-specific deletion of the *Pnoc* gene. Littermate controls (*Pnoc*^wt/wt^::*Cd19*Cre^tg/wt^) carried the Cd19-Cre transgene but lacked the floxed *Pnoc* allele. PCR analysis confirmed the recombined (knockout) allele in splenic tissue from *Pnoc*ΔCD19 mice (Figure S1E). Furthermore, qPCR analysis demonstrated markedly reduced *Pnoc* mRNA expression in both whole spleen tissue (Figure 1E) and isolated splenic B cells (Figure 1F). To assess whether *Pnoc* deletion affects spleen morphology under basal conditions, we measured spleen length (Figure 1G) and weight (Figure 1H) in *Pnoc*ΔCD19 and controls. No notable differences were observed, indicating that B cell-specific *Pnoc* deletion does not impact spleen development.

### Metabolic profiling of B cell-specific *Pnoc* knockout mice reveals mildly enhanced insulin sensitivity without changes in glucose tolerance

**Figure 2:**
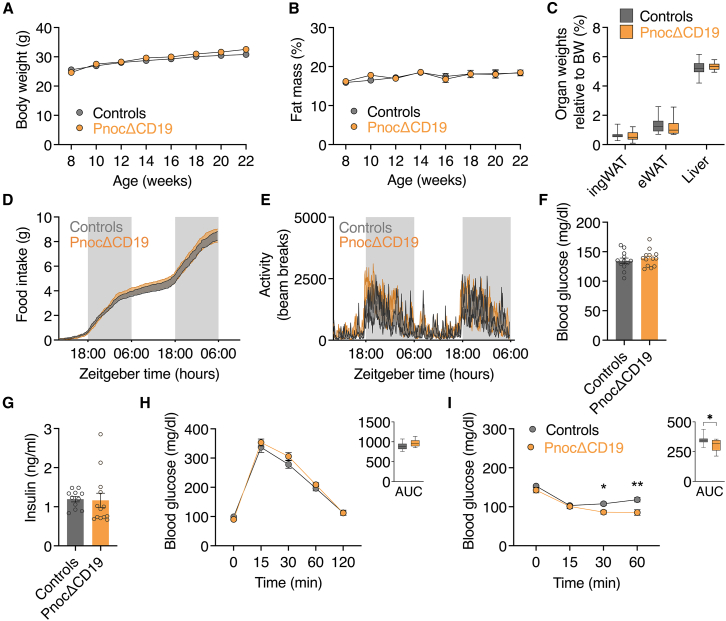
Metabolic profiling of B cell-specific *Pnoc* knockout mice reveals mildly enhanced insulin sensitivity without changes in glucose tolerance (A) Body weight of control (*n* = 12) and *Pnoc*ΔCD19 (*n* = 12) mice, measured weekly from 8 to 22 weeks of age. (B) Percentage fat mass of control (*n* = 12) and *Pnoc*ΔCD19 (*n* = 12) mice measured every two weeks from 8 to 22 weeks of age. (C) Relative tissue weight of inguinal white adipose tissue (ingWAT), epididymal white adipose tissue (eWAT), and liver from control (*n* = 12) and *Pnoc*ΔCD19 (*n* = 12) mice at 24 weeks of age. (D) *Ad libitum* food intake measured during two dark and two light cycles in control (*n* = 11) and *Pnoc*ΔCD19 mice (*n* = 11). (E) Voluntary physical activity, measured by beam breaks count, was recorded during two dark and two light cycles in control (*n* = 11) and *Pnoc*ΔCD19 mice (*n* = 11). (F) Blood glucose levels measured at 24 weeks of age in control (*n* = 11) and *Pnoc*ΔCD19 (*n* = 11) mice. (G) Serum insulin levels measured at 24 weeks of age in control (*n* = 11) and *Pnoc*ΔCD19 (*n* = 11) mice. (H) Glucose tolerance tests (GTTs) performed in control (*n* = 11) and *Pnoc*ΔCD19 (*n* = 11) mice between 14 and 16 weeks of age. Blood glucose levels were measured at the indicated time points following glucose administration. The area under the curve (AUC) is presented in the right panel. (I) Insulin tolerance tests (ITTs) performed on control (*n* = 12) and *Pnoc*ΔCD19 (*n* = 12) mice between 14 and 16 weeks of age. Blood glucose levels were measured at the indicated time points following insulin administration. Area under the curve (AUC) is presented in the right panel. Data are presented as mean ± SEM. Statistical analyses were performed by two-tailed Student’s t test (C–E) and two-way ANOVA followed by Sidak’s multiple comparisons test (A, B and F, G). Significance levels are indicated as ∗*p* < 0.05, ∗∗*p* < 0.01, and ∗∗∗*p* < 0.001.

Metabolic characterization of mice maintained on a standard chow diet revealed no differences between *Pnoc*ΔCD19 mice and controls in terms of body weight (Figure 2A), fat mass assessed by nuclear magnetic resonance (NMR) (Figure 2B), as well as organ weights (Figure 2C). Food intake and activity levels were also comparable (Figures 2D and 2E), with no observed changes in respiratory exchange ratio (RER) or energy expenditure (Figures S2A and S2B). Baseline blood glucose and insulin levels did not differ between *Pnoc*ΔCD19 mice and controls (Figures 2F and 2G). Glucose tolerance tests (GTTs) conducted at 16 weeks of age showed no notable differences between the groups (Figure 2H). Interestingly, however, insulin tolerance tests (ITTs) at 14 weeks revealed a modest improvement in insulin sensitivity in *Pnoc*ΔCD19 mice (Figure 2I). Given this increase in insulin sensitivity, despite otherwise unchanged metabolic parameters, we next investigated whether conditional *Pnoc* deletion in B cells could mitigate the development of high-fat diet (HFD)-induced insulin resistance.

### B cell-specific *Pnoc* deletion enhances glucose tolerance and insulin sensitivity during high-fat diet feeding

**Figure 3:**
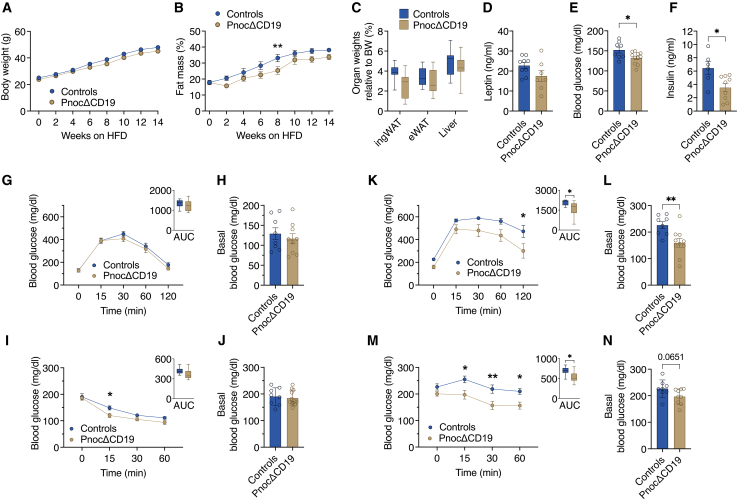
B cell-specific *Pnoc* deletion enhances glucose tolerance and insulin sensitivity during high-fat diet feeding (A) Body weight of control (*n* = 8) and *Pnoc*ΔCD19 (*n* = 11) mice after HFD feeding. (B) Percentage fat mass of control (*n* = 8) and *Pnoc*ΔCD19 (*n* = 11) mice fed an HFD, measured every two weeks. (C) Relative tissue weight of inguinal white adipose tissue (ingWAT), epididymal white adipose tissue (eWAT), and liver from control (*n* = 8) and *Pnoc*ΔCD19 (*n* = 11) mice after 16 weeks of HFD feeding. (D) Serum leptin levels in control (*n* = 8) and *Pnoc*ΔCD19 (*n* = 7) mice after 16 weeks of HFD feeding. (E) Blood glucose levels in control (*n* = 8) and *Pnoc*ΔCD19 (*n* = 11) mice after 16 weeks of HFD feeding. (F) Serum insulin levels in control (*n* = 8) and *Pnoc*ΔCD19 (*n* = 11) mice after 16 weeks of HFD feeding. (G) Glucose tolerance test (GTT) performed after 8 weeks of HFD in control (*n* = 8) and *Pnoc*ΔCD19 (*n* = 11) mice. Blood glucose levels were measured at the indicated time points following glucose administration. Area under the curve (AUC) is shown in the right panel. (H) Baseline blood glucose levels measured after overnight fasting in control (*n* = 8) and *Pnoc*ΔCD19 (*n* = 10) mice. (I) Insulin tolerance test (ITT) performed after 6 weeks of HFD in control (*n* = 8) and *Pnoc*ΔCD19 (*n* = 11) mice. Blood glucose levels were measured at the indicated time points following insulin administration. Area under the curve (AUC) is shown in the right panel. (J) Baseline blood glucose levels measured prior to insulin injection during the ITT in control (*n* = 8) and *Pnoc*ΔCD19 (*n* = 11) mice. (K) GTTs performed after 14 weeks of HFD in control (*n* = 8) and *Pnoc*ΔCD19 (*n* = 10) mice. (L) Baseline blood glucose levels measured after overnight fasting in control (*n* = 8) and *Pnoc*ΔCD19 (*n* = 10) mice. (M) ITTs performed after 12 weeks of HFD in control (*n* = 8) and *Pnoc*ΔCD19 (*n* = 11) mice. Blood glucose levels were measured at the indicated time points following insulin administration. Area under the curve (AUC) is shown in the right panel. (N) Baseline blood glucose levels measured in control (*n* = 8) and *Pnoc*ΔCD19 (*n* = 9) mice. Data are presented as mean ± SEM. Statistical analyses were performed using two-way ANOVA followed by Sidak’s multiple comparisons test (A, B, G, I, K, and M) and two-tailed Student’s t test (C, D, E, F, H, J, L, and N as well as the AUC inserts in G, K, I, and M). Significance levels are indicated as ∗*p* < 0.05, ∗∗*p* < 0.01, and ∗∗∗*p* < 0.001.

Therefore, starting at 8 weeks of age, we challenged *Pnoc*ΔCD19 mice and their littermate controls with an HFD containing 60% kcal from fat. Throughout the study, body weight gain was comparable between the two groups (Figure 3A). Consistent with this finding, analyses of RER and energy expenditure revealed no differences between *Pnoc*ΔCD19 and control mice (Figures S3A and S3B). Interestingly, NMR measurements revealed a trend toward reduced fat mass in *Pnoc*ΔCD19 mice, particularly noticeable 8 weeks after the initiation of the HFD. This effect diminished in subsequent weeks (Figure 3B). After 16 weeks of HFD, no notable differences were observed in the weights of inguinal (ingWAT) and epididymal (eWAT) adipose tissue depots or the liver (Figure 3C). Importantly, spleen weight and length remained unchanged throughout the HFD feeding period (Figures S3C and S3D). Corresponding to the similar adiposity levels, leptin serum levels were unchanged between the groups (Figure 3D). Importantly, significant differences were observed in fed basal blood glucose and insulin levels, with *Pnoc*ΔCD19 mice exhibiting markedly decreased levels compared to controls (Figures 3E and 3F).

To investigate the impact of *Pnoc*ΔCD19 deletion on glycemic control and insulin sensitivity, we performed GTTs and ITTs at various time points during HFD exposure. GTTs at the intermediary stage of HFD feeding (6 weeks) showed no differences in glucose tolerance or basal blood glucose between groups (Figure 3G and 3H). However, *Pnoc*ΔCD19 mice exhibited a slight reduction in blood glucose levels 15 min into the ITT conducted after 4 weeks of HFD, although basal glucose levels remained similar (Figures 3I and 3J). After long-term feeding of HFD for 16 weeks, GTT results indicated improved glucose tolerance in *Pnoc*ΔCD19 mice, alongside lower basal blood glucose levels (Figures 3K and 3L). Insulin tolerance was also markedly improved in *Pnoc*ΔCD19 mice after 14 weeks of HFD (Figure 3M), with a trend toward lower basal blood glucose in random-fed conditions before the ITT (Figure 3N). Collectively, our experiments demonstrate that conditional deletion of *Pnoc* in B cells confers protection against HFD-induced insulin resistance and impaired glucose tolerance, while maintaining normal body weight regulation.

### B cell-specific *Pnoc* deletion alters immune cell recruitment in the liver under high-fat diet conditions

**Figure 4:**
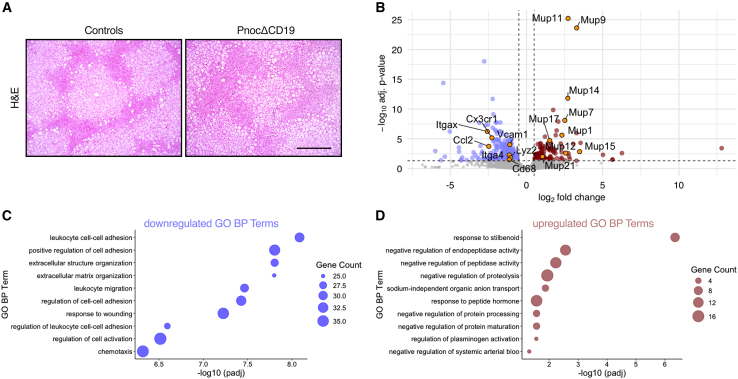
B cell-specific *Pnoc* deletion alters immune cell recruitment in the liver under high-fat diet conditions (A) Representative images of H&E-stained liver sections from control and *Pnoc*ΔCD19 mice fed a HFD. Scale bar: 200 μm. (B) Volcano plot illustrating the distribution of differentially expressed genes in liver samples from control (*n* = 3) and PnocΔCD19 (*n* = 3) mice fed an HFD. (C) Gene ontology (GO) term analysis highlighting downregulated signaling pathways (dark blue) in liver samples from PnocΔCD19 mice compared to controls. (D) GO term analysis highlighting upregulated signaling pathways (dark red) in liver samples from PnocΔCD19 mice compared to controls.

To further explore the mechanistic underpinnings of the improved glucose tolerance and insulin sensitivity observed in *Pnoc*ΔCD19 mice on HFD, we investigated how B cell-specific *Pnoc* deletion influences signaling in key metabolic organs, such as liver and adipose tissue. First, we sought to validate the expression of *PNOC* mRNA in human liver-associated B cells. To do this, we analyzed a publicly available single-cell RNA-sequencing dataset (GEO accession GSE136103) comprising CD45^+^ and CD45^−^ immune cells from liver tissue samples of healthy human donors (45). These data confirm that *PNOC* is specifically expressed in hepatic B cells (Figure S4A). Building on this observation, we next examined liver tissue from HFD-fed *Pnoc*ΔCD19 and control mice. H&E stainings did not reveal any gross morphological abnormalities (Figure 4A), and hepatic triglyceride levels were comparable between the two groups (Figure 4B). However, CD86 immunostaining suggested a visible reduction in macrophage abundance in the livers of *Pnoc*ΔCD19 mice under HFD conditions (Figure S4C). Subsequent RNA sequencing and differential gene expression analysis identified 443 differentially expressed genes in liver samples of HFD-fed *Pnoc*ΔCD19 mice compared to controls (Figure 4B). Gene set enrichment analysis (GSEA) of the downregulated genes revealed significant enrichment for biological processes related to immune and inflammatory responses, including “extracellular matrix organization”, “leukocyte migration”, “leukocyte cell-cell adhesion”, and “chemotaxis”, among others (Figure 4C). Specifically, we observed downregulation of *Cx3cr1* (C-X3-C motif chemokine receptor 1), *Itga4* (integrin alpha 4), *Vcam1* (vascular cell adhesion molecule 1), *Cd68* (cluster of differentiation 68), *Lyz2* (lysozyme 2), *Itgax* (integrin alpha X), and *Ccl2* (C-C motif chemokine ligand 2). These genes are integral to macrophage chemotaxis, adhesion, and migration of pro-inflammatory macrophages into the liver. qPCR analysis revealed an overall reduction in the expression of the pan-macrophage marker F4/80 (*Adgre1*), while no changes were detected in macrophage polarization markers such as *Nos2* or *Arg1* (Figures S4D–S4F). In contrast, the upregulated genes were enriched for processes associated with the Gene Ontology (GO) term “response to stilbenoid” (Figure 4D). Specifically, our analysis uncovered marked upregulation of a cluster of genes of the major urinary protein (MUP) family, including *Mup1*, *Mup3*, *Mup7*, *Mup9*, *Mup11*, *Mup12*, *Mup14*, *Mup15*, *Mup17*, and *Mup21* (Figure 4B). MUP proteins are predominantly synthesized and secreted by hepatocytes and have been implicated in the modulation of metabolic homeostasis, glucose and lipid metabolism.^46^ Given that immune cell infiltration into adipose tissue is a hallmark of diet-induced obesity, we next examined visceral adipose tissue depots of HFD-fed *Pnoc*ΔCD19 and control mice.

### B cell-specific *Pnoc* deletion alters macrophage recruitment and improves visceral adipose health under high-fat diet conditions

**Figure 5:**
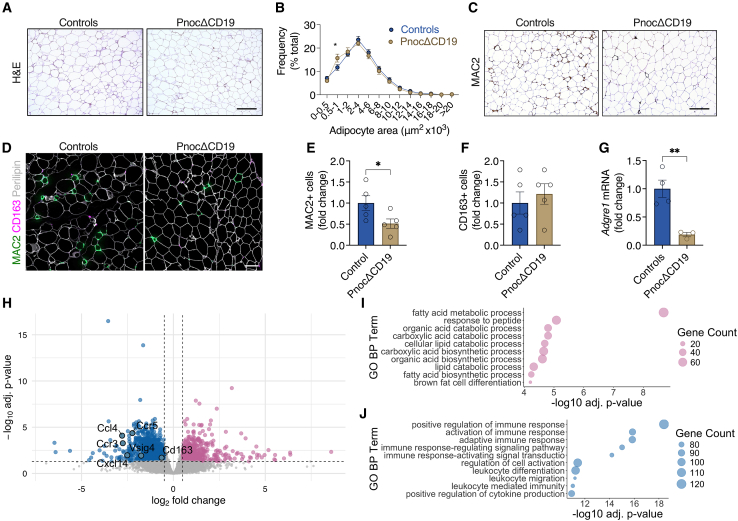
B cell-specific *Pnoc* deletion alters macrophage recruitment and improves visceral adipose health under high-fat diet conditions (A) Representative images of H&E staining of epididymal white adipose tissue (eWAT) from control and *Pnoc*ΔCD19 mice fed an HFD. Scale bar: 200 μm. (B) Quantification of adipocyte area in eWAT samples from control and *Pnoc*ΔCD19 mice fed an HFD. (C) Representative images of immunohistochemical staining for MAC2 in epididymal white adipose tissue (eWAT) from control and *Pnoc*ΔCD19 mice fed an HFD. Scale bar: 200 μm. (D) Immunofluorescence staining of MAC2 (green), CD163 (magenta) and Perilipin (white). Scale bar: 100 μm. Cells were counted in four distinct fields of the same section per sample, and the average number per section was calculated. (E) Quantification of MAC2-expressing cells eWAT from control (*n* = 5) and *Pnoc*ΔCD19 (*n* = 5) mice fed an HFD. (F) Quantification of CD163-expressing cells eWAT from control (*n* = 5) and *Pnoc*ΔCD19 (*n* = 5) mice fed an HFD. (G) Validation of the pan-macrophage marker *Adgre1* (F4/80, Emr1) expression by RT-qPCR in eWAT samples from control (*n* = 4) and *Pnoc*ΔCD19 (*n* = 3) mice fed an HFD. (H) Volcano plot depicting the distribution of differentially expressed genes in eWAT samples from control (*n* = 4) and *Pnoc*ΔCD19 (*n* = 3) mice fed an HFD. (I and J) Gene ontology (GO) term analysis highlighting (I) upregulated (purple) and (J) downregulated (blue) signaling pathways in eWAT samples from control (*n* = 4) and *Pnoc*ΔCD19 (=3) mice fed an HFD. Data are presented as mean ± SEM. Statistical analyses were performed using two-way ANOVA followed by Sidak’s multiple comparisons test (B) or two-tailed Student’s t test (E, F, and G). Significance levels are indicated as ∗*p* < 0.05 and ∗∗*p* < 0.01.

H&E staining of eWAT from HFD-fed mice indicated smaller adipocytes and a reduction in crown-like structures (CLS) in *Pnoc*ΔCD19 mice (Figure 5A). Analysis of adipocyte size distribution confirmed a marked increase in the proportion of smaller adipocytes (Figure 5B). Notably, a comparable shift toward smaller adipocyte populations was also observed in the ingWAT of HFD-fed *Pnoc*ΔCD19 mice (Figures S5A and S5B). Furthermore, analysis of MAC2-expressing adipose tissue macrophages demonstrated a notable reduction in inflammatory macrophage content in HFD-fed *Pnoc*ΔCD19 mice compared to controls (Figures 5C–5E). In contrast, no differences were detected in the abundance of CD163-expressing macrophages, a marker of M2-like macrophages (Figure 5F). These findings were corroborated by quantitative PCR, which showed reduced expression of *Adgre1* (F4/80, Emr1) mRNA in eWAT of *Pnoc*ΔCD19 mice (Figure 5G). A similar decrease in *Adgre1* expression was observed in ingWAT, whereas expression levels of the M1-associated marker *Nos2* and the M2-associated marker *Arg1* remained unchanged (Figures S5C–S5E). Our findings align with previous studies indicating that inflammation within subcutaneous adipose tissue contributes to systemic insulin resistance and metabolic dysfunction in obesity.^47^ To further elucidate the molecular mechanisms underlying these phenotypic changes, we performed RNA sequencing analysis on eWAT samples from HFD-fed mice. This analysis identified 1,897 differentially expressed genes (Figure 5H). GSEA of the downregulated genes revealed an enrichment of gene signatures associated with immune responses and cell migration. Specifically, downregulated genes were enriched in pathways related to “positive regulation of immune response”, “leukocyte migration”, and “positive regulation of cytokine production”, among others (Figure 5J). Interestingly, the GSEA revealed that the upregulated gene signatures in adipose tissue were predominantly related to metabolic improvements. Specifically, pathways such as “fatty acid metabolic process”, “fatty acid biosynthetic process”, and “brown fat cell differentiation” were upregulated (Figure 5I). The observed changes in gene expression reveal a decrease in the inflammatory state within visceral adipose tissue, which aligns with the improvements in insulin sensitivity of *Pnoc*ΔCD19 mice. Moreover, comparing RNA sequencing data from adipose and liver tissues revealed a global reduction in inflammatory recruitment pathways. *Cx3cr1* and *Itga4*, key mediators of monocyte/macrophage recruitment and lymphocyte extravasation, were downregulated in both tissues. *Vcam1*, essential for leukocyte-endothelial adhesion, was similarly reduced, indicating a broad suppression of immune cell migration in *Pnoc*ΔCD19 mice.

### B cell-specific Pnoc deletion attenuates systemic chemokine expression and N/OFQ-mediated macrophage recruitment

**Figure 6:**
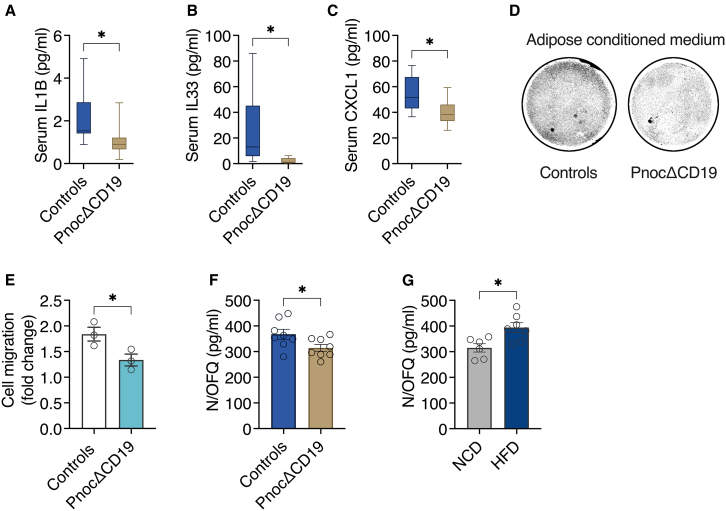
B cell-specific Pnoc deletion attenuates systemic chemokine expression and N/OFQ-mediated macrophage recruitment (A–C) Serum concentrations of IL-1β (A), IL-33 (B), and CXCL1 (C) measured by OLINK targeted proteomics in HFD-fed control (*n* = 8) and PnocΔCD19 (*n* = 10) mice. (D and E) Representative images (D) and bar graph showing fold change in cell migration from transwell migration assays (E). BMDMs were treated for 24 h with adipose tissue-conditioned media from control or *Pnoc*ΔCD19 mice (*n* = 4). (F) Serum N/OFQ levels in control (*n* = 8) and *Pnoc*ΔCD19 (*n* = 8) mice fed an HFD. (G) Serum N/OFQ levels in mice fed a normal chow diet (NCD) (*n* = 6) or an HFD (*n* = 7). Data are presented as mean ± SEM. Statistical analyses were performed using two-tailed Student’s t test (A, B, C, E, F, and G). Significance levels are indicated as ∗*p* < 0.05.

To assess how B cell-specific *Pnoc* deletion influences systemic inflammatory signaling, we employed OLINK serum proteomics. This analysis revealed a marked reduction in serum levels of interleukin-1β (IL-1β), IL-33, and CXCL1 in HFD-fed *Pnoc*ΔCD19 mice relative to controls (Figures 6A–6C). We next examined whether the adipose tissue secretome of *Pnoc*ΔCD19 mice modulates macrophage recruitment. Therefore, we prepared adipose tissue-conditioned medium from *Pnoc*ΔCD19 and control mice. In transwell migration assays using bone marrow-derived macrophages (BMDMs), the conditioned medium from *Pnoc*ΔCD19 adipose tissue markedly reduced macrophage migration compared to controls (Figures 6D and 6E). This finding indicates that *Pnoc* deficiency in B cells alters the adipose tissue secretome, thereby limiting macrophage recruitment. Consistent with this, we observed lower circulating levels of N/OFQ in HFD-fed *Pnoc*ΔCD19 mice than in HFD-fed controls (Figure 6F). In line with this finding, independent experiments revealed that N/OFQ levels are increased in wild-type mice during HFD feeding compared to animals on a normal chow diet (NCD) (Figure 6G).

### N/OFQ enhances macrophage migration and bioenergetic phenotype through receptor-mediated chemotactic signaling

**Figure 7:**
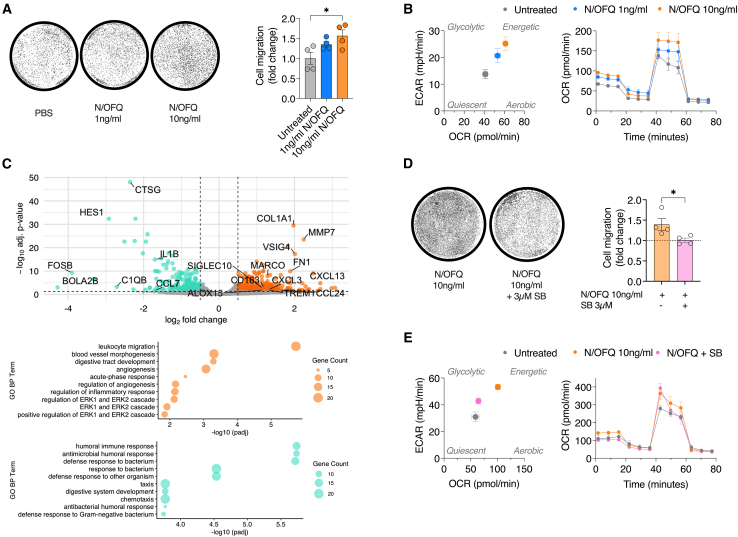
N/OFQ enhances macrophage migration and bioenergetic phenotype through receptor-mediated chemotactic signaling (A) Representative images and quantification of fold change in cell migration from transwell migration assays performed on differentiated THP-1 macrophages treated with 1 ng/mL or 10 ng/mL recombinant N/OFQ for 24 h (*n* = 4). (B) Seahorse analysis of THP-1 differentiated macrophages treated for 24 h with 1 ng/mL or 10 ng/mL of N/OFQ (*n* = 4). Baseline oxygen consumption rate (OCR) as measure for respiration was plotted against the extracellular acidification rate (ECAR) as measure for glycolysis. OCR measurements were recorded during the Mito Stress Seahorse assay following the sequential administration of oligomycin, FCCP, and antimycin/rotenone. (C) Volcano plot showing the distribution of differentially expressed genes in differentiated macrophages treated for 24 h with 10 ng/mL N/OFQ. Gene ontology (GO) term analysis highlighting downregulated (green) and upregulated (orange) signaling pathways in differentiated macrophages treated for 24 h with 10 ng/mL N/OFQ. (D) Representative micrographs and quantification of THP-1-derived macrophage migration in transwell assays following 24-h stimulation with 10 ng/mL N/OFQ, with or without co-treatment using 3 μM SB-612111 (*n* = 4). (E) Seahorse analysis of differentiated THP-1 macrophages treated for 24 h with 10 ng/mL N/OFQ and co-treated with 3 μM SB-612111 (*n* = 4). Data are presented as mean ± SEM. Statistical analyses were performed using two-tailed Student’s t test. Significance levels are indicated as ∗*p* < 0.05, ∗∗*p* < 0.01, and ∗∗∗*p* < 0.001.

To investigate the effect of N/OFQ on macrophages, we differentiated the human monocytic cell line THP-1 using phorbol 12-myristate 13-acetate (PMA) and subsequently treated the cells with 1 ng/mL and 10 ng/mL of recombinant N/OFQ. Transwell migration assays showed that a 24-h incubation with 10 ng/mL N/OFQ significantly promoted THP-1 macrophage migration compared to untreated controls (Figure 7A). Since immune cell migration requires dynamic cytoskeletal rearrangement, which is inherently energy demanding,^48^ we analyzed the cellular bioenergetic profile of these cells using an Agilent Seahorse XF Analyzer (Figure 7B). We observed that N/OFQ treatment shifted the bioenergetic metabolism of macrophages to a more energetic phenotype, as demonstrated by elevated oxygen consumption rate (OCR) and extracellular acidification rate (ECAR) in macrophages treated for 24 h with 10 ng/mL N/OFQ (Figure 7B). We extended our analysis to undifferentiated monocytic THP-1 cells and found these cells to be even more responsive to N/OFQ stimulation. A pronounced migratory response was already evident at a concentration as low as 1 ng/mL (Figure S6A). Consistent with our observations in differentiated macrophages, N/OFQ treatment also induced a metabolic shift toward a more energetic phenotype in these undifferentiated cells, as reflected by elevated oxidative and glycolytic activity (Figure S6B). In contrast, exposure to 100 ng/mL LPS shifted the bioenergetic profile of THP-1 cells toward a predominantly glycolytic state, indicative of a classical inflammatory activation pattern (Figure S6B). Importantly, the observed metabolic changes were not associated with alterations in the respiratory chain stoichiometry (Figure S6C). Subsequent RNA sequencing analysis of THP-1 macrophages treated with 10 ng/mL N/OFQ for 24 h compared to untreated controls revealed a total of 245 differentially regulated genes (Figures 7C and S6D). GSEA identified “leukocyte migration” as the only significantly upregulated gene set among these genes (Figure 7C). Notably, the differentially expressed genes associated with this term included *COL1A1*, *FN1*, *EDNRB*, *SLC16A8*, *SIRPG*, *CXCL13*, *ANGPT4*, *CD48*, *APOD*, *TREM1*, *CXCL3*, and *CCL24*. Conversely, the downregulated genes were enriched in gene sets related to immune and inflammatory responses. These included pathways such as “antibacterial humoral response”, “defense response to other organism”, “humoral immune response”, and “response to bacterium” (Figure 7C). Collectively, our data suggest that N/OFQ enhances gene expression profiles associated with the migratory capacity of macrophages.

To determine whether these effects were mediated through the NOP receptor, we co-treated THP-1 macrophages with 10 ng/mL N/OFQ and SB-612111, a selective NOP receptor antagonist. 3 μM SB-612111 effectively inhibited N/OFQ-induced macrophage migration (Figure 7D), confirming the receptor-dependent nature of the migratory response. Moreover, the enhanced bioenergetic metabolism observed with N/OFQ treatment was reversed by SB-612111 (Figure 7E). Overall, these findings demonstrate that N/OFQ promotes macrophage migration by activating key chemotactic signaling pathways, an effect that is directly mediated through NOP receptor engagement.

## Discussion

Our findings demonstrate that a B cell-specific conditional *Pnoc* knockout improves glycemic control and enhances insulin sensitivity in the context of diet-induced obesity, despite comparable adiposity. These findings align with previous studies demonstrating that B cell-deficient obese mice exhibit comparable body weight to control animals but display lower fasting glucose levels and enhanced glucose tolerance.^42^ Additionally, B cell deficiency in obese mice is associated with reduced proinflammatory cytokine production in adipose tissue and reconstitution of these mice with splenic B cells derived from obese donors reverses these benefits, leading to impaired glucose tolerance.^42^ Similarly, DeFuria and colleagues demonstrated that ablation of B cells enhances glucose tolerance in obese mice without altering body weight, likely through reduced B cell-mediated proinflammatory signaling that attenuates T cell-driven inflammation in adipose tissue.^41^ B cells infiltrate adipose tissue early during the onset of diet-induced obesity, where they can influence inflammation and metabolic dysfunction.^43^ In obese individuals with type 2 diabetes (T2D), B cells exhibit a more pronounced inflammatory cytokine expression profile, contributing to the promotion of T cell-mediated inflammation.^41^^,^^49^ Furthermore, these cells exhibit impaired regulatory function, failing to adequately upregulate IL-10 production. Within visceral adipose tissue, B-1a cells serve as the primary source of B cell-derived IL-10, contributing nearly half of its total production. However, in obese mice fed an HFD, the number of B-1a cells is notably reduced, resulting in a decline in IL-10 production.^50^ B cells produce different antibody isotypes with distinct functions. While IgG antibodies contribute to metabolic deterioration, IgM antibodies, especially those from B-1b cells, offer protective effects.^51^ Conversely, previous studies have shown that IgG antibodies can exacerbate adipose tissue inflammation and promote insulin resistance in obesity.^42^ Given the functional dichotomy between B-1 and B-2 cell subsets in metabolic regulation, investigating how *Pnoc* expression is distributed across these lineages, and how it influences their function, remains a key area for future research. It is also yet to be determined whether *Pnoc* expression influences immunoglobulin class switching or modulates the effector phenotype of antibody-secreting cells in the context of metabolic inflammation.

In our analysis of over 1,480 human visceral adipose tissue biopsies, we identified *PNOC*-expressing B cells within the visceral adipose tissue of obese patients. Notably, *PNOC* expression in B cells has been previously identified within the tumor microenvironment of cholangiocarcinoma, where it is associated with increased immune infiltration and positively correlated with improved patient survival outcomes. Here, *PNOC* was specifically expressed in infiltrating B cells, including naive, activated, and plasma cell subsets, based on single-cell RNA-seq analysis.^52^ *Pnoc*ΔCD19 mice on HFD showed a notable reduction in markers of inflammation within liver and eWAT. GSEA of eWAT RNA-seq data revealed a broad downregulation of pathways associated with immune cell migration and leukocyte adhesion. Notably, key mediators of monocyte and macrophage trafficking to inflamed tissues, such as *Cx3cr1* and *Itga4*, were markedly downregulated.^53^^,^^54^ This reduction in pro-inflammatory signaling likely contributes to the observed improvements in insulin sensitivity and glucose tolerance. The marked upregulation of multiple MUP genes in livers of *Pnoc*ΔCD19 mice under HFD conditions could provide a mechanistic link to the improved glucose and insulin tolerance. MUPs, particularly MUP1, are hepatokines known to enhance systemic insulin sensitivity and promote glucose oxidation in peripheral tissues, partly via increased mitochondrial oxidative capacity in skeletal muscle.^55^ Moreover, MUP1 has been shown to suppress hepatic gluconeogenesis independently of insulin signaling, indicating direct effects on liver glucose metabolism.^56^

In our study, treatment of THP-1 macrophages with recombinant N/OFQ induced the expression of CXCL13, a chemokine critical for recruiting B cells to sites of inflammation.^57^^,^^58^ Vice versa, stimulation of B cells with CXCL13 (and IL-4) promotes the release of N/OFQ.^38^ Notably, N/OFQ has been shown to exert a direct inhibitory effect on B cell function by suppressing antibody production in response to antigenic stimulation, indicating that B cell-derived N/OFQ could act in an autocrine or paracrine manner to modulate immune responses within the B cell compartment itself.^59^ Additionally, inflammatory activators such as PMA increase NOP receptor expression in THP-1 macrophages.^60^ Taken together, these findings indicate a feedback loop where N/OFQ promotes crosstalk between B cells and macrophages, influencing immune cell migration and cytokine release within adipose tissue.

To determine whether the effects observed are dependent on the NOP receptor, we used SB-612111, a potent NOP receptor antagonist.^61^ SB-612111 exhibits high selectivity for the NOP receptor, showing negligible activity at μ-, δ-, and κ-opioid receptors, as well as minimal affinity for adrenergic and H2-histamine receptors.^61^^,^^62^ Treatment with SB-612111 effectively inhibited N/OFQ-induced migration of THP-1-derived macrophages and reversed the associated enhancement in the bioenergetic state of the cells, conclusively demonstrating that these responses are mediated through the NOP receptor. However, SB-612111 exhibits central nervous system penetrance and has been shown to reduce binge eating and overall food intake through centrally mediated mechanisms in rodents.^63^ Given the role of central N/OFQ signaling in regulating feeding behavior, it is challenging to distinguish whether any metabolic improvements observed with SB-612111 treatment arise from its peripheral anti-inflammatory effects or from reduced caloric intake due to central appetite suppression. Here, peripherally restricted NOP antagonists could be used to reduce overall metabolic inflammation and improve glucose homeostasis without affecting central appetite regulation.

Our findings emphasize the role of immune cell-derived opioid peptides, such as N/OFQ, in regulating macrophage function within adipose tissue, particularly in the context of metabolic inflammation associated with obesity and type 2 diabetes. Targeting the NOP receptor with antagonists may modulate immune responses and improve metabolic outcomes in these conditions. However, further research is warranted to investigate the therapeutic potential of NOP receptor antagonism in metabolic diseases.

### Limitations of the study

This study has several limitations. The dynamic regulation of *PNOC* expression in B cells in human obesity was not assessed, owing to the lack of single-cell datasets with sufficient B cell representation across metabolic states. Tissue-specific insulin signaling was not evaluated; quantification of insulin-stimulated AKT phosphorylation in liver and adipose tissue would help identify the site of improved insulin sensitivity. Hepatic gluconeogenesis was not directly tested, and the contribution of individual MUPs to glucose homeostasis remains undefined, despite the correlation between hepatic MUP induction and improved glucose regulation.

## Resource availability

### Lead contact

Further information and requests for resources and reagents should be directed to and will be fulfilled by the lead contact, Alexander Jais (alexander.jais@helmholtz-munich.de).

### Materials availability

Requests for materials and reagents should be directed to the lead contact.

### Data and code availability

• Raw and fully processed RNA-seq data are available at GEO accession number GSE281815 for eWAT, GSE281911 for liver tissue, and GSE281913 for THP-1 macrophages. The human RNA-seq data from the LOBB have not been deposited in a public repository due to restrictions imposed by patient consent but can be obtained from M.B. upon request.
• This study did not generate original code.
• Any additional information required to reanalyze the data reported in this paper is available from the lead contact upon request.

## Acknowledgments

We wish to thank Anja Moll, Jenny Schuster, Lisa Gärtner, Elisabeth Langer, Anja Recknagel, and Florian Geier for excellent technical assistance. Furthermore, we thank Dr. Konstanze Julich-Gruner and Susanne Renno for administrative help. A.J. received funding by the 10.13039/501100001659German Research Foundation (DFG) (537071551) and the 10.13039/501100001648EFSD/10.13039/501100009708Novo Nordisk Foundation (NNF22SA0081231, 10.13039/501100001648EFSD/10.13039/501100009708Novo Nordisk Foundation Future Leaders Award). This work was supported by the Helmholtz Center Munich and the federal government of Saxony, Germany.

## Author contributions

Conceptualization, A.J. and S.C.P.-R.; methodology: S.CP.-R., L.I., J.S., A.B.-K., A.H., A.G., F.N.; investigation, S.C.P.-R., L.I., J.S., A.B.-K., and A.H.; formal analysis, S.C.P.-R, L.I., and A.J.; visualization, S.C.P.-R., A.H., and A.J.; manuscript, A.J.; editing, S.C.P.-R, J.S., and A.J.; project administration, A.J.; resources, C.W., K.K., M.G., N.K., J.C.B., F.T.W., M.B., and A.J.

## Declaration of interests

M.B. has received honoraria as a consultant from Amgen, AstraZeneca, Bayer, Boehringer-Ingelheim, Lilly, Novo Nordisk, Novartis, and Sanofi. J.C.B. is a cofounder of Cerapeutix and has received honoraria as consultant from Eli Lilly and Company and Novo Nordisk.

## STAR★Methods

### Key resources table

**Table:** 

REAGENT or RESOURCE	SOURCE	IDENTIFIER
**Critical commercial assays**		

RPMI 1649 medium (ATCC-Modification)	Gibco	Cat# A1049101
Fetal bovine serum	Sigma	Cat# F7524
Penicillin/streptomycin	VWR	Cat# SIALP4333
Trypsin-EDTA (0.25%), phenol red	Thermo Fisher Scientific	Cat# 25200056
Phorbol 12-myristate 13-acetate	Sigma	Cat# P8139
Red blood cell lysis buffer	Invitrogen	Cat# 00-4333-57
Seahorse XF calibrant buffer	Agilent	Cat# 00840-000
D-(+)-Glucose solution, 45%	Sigma	Cat# G8769
L-Glutamine solution (200 mM)	Sigma	Cat# G7513
Sodium pyruvate solution (100 mM)	Sigma	Cat# S8636
Seahorse XF base medium	Agilent	Cat# 102353-100
DAPI for nucleic acid staining	Sigma-Aldrich	Cat# D9542
Low ROX SYBR 2X MasterMix blue dTTP	Eurogentec	Cat# UF-LSMT-B0701
CellTak	Corning	Cat# 354240
RIPA buffer	ThermoFisher	Cat# 89901
Protease Inhibitor Cocktail (100X)	Cell Signaling Technology	Cat# 5871S
Protease/Phosphatase Inhibitor Cocktail (100X)	Cell Signaling Technology	Cat# 5872S
Western blotting membranes, nitrocellulose, 0.2μm	Merck	Cat# 10600053
Pierce BCA Protein Assay Kit	Thermo Fisher Scientific	Cat# 23227
Pierce ECL Western Blotting Substrate	VWR	Cat# PIER32109
N/OFQ ELISA Kit	Elabscience	Cat# E-EL-M0862
Ultrasensitive Mouse Insulin ELISA Kit	Crystal Chem	Cat# 90080
Mouse Leptin ELISA Kit	Crystal Chem	Cat# 90030
Seahorse XF MitoStress Kit	Agilent	Cat# 103015-100
Pan B Cell Isolation Kit	Miltenyi Biotec	Cat# 130-121-301
RNeasy Lipid Tissue Kit	Qiagen	Cat# 74804
High-Capacity cDNA Reverse Transcription Kit	Life Technologies	Cat# 4368814
HRP/DAB Detection IHC Kit	Abcam	Cat# ab64264b
Triglyceride Assay Kit	Abcam	Cat# ab65336
Seahorse XFe96 FluxPak	Agilent	Cat# 102416-100
RNeasy Mini Kit	Qiagen	Cat# 5001329
Corning FluoroBlok 24-multiwell insert	VWR	Cat# 734-0371
Millicell 24 well Plate (8.0 μm pore size)	Merck	Cat# PSET010
TrueBlack Lipofuscin Autofluorescence Quenche	Biotium	Cat# 23007
Olink Target 48 Mouse	Olink	Cat# 93400

**Antibodies**		

MAC-2 and Gal-3 (Galectin-3) Monoclonal antibody, Unconjugated, Clone m3/38	Cedarlane	Cat# CL8942AP; RRID:AB_10060357
CD86	Cell Signaling Technology	Cat# 19589; RRID:AB_2892094
CD163	Abcam	Cat# ab182422; RRID:AB_2753196
Perilipin	Abcam	Cat# ab61682; RRID:AB_944751
Donkey anti-Rat IgG (H + L) Cross-Adsorbed Secondary Antibody, DyLight™ 488	Invitrogen	Cat# SA5-10026; RRID:AB_2556606
Donkey anti-Goat IgG (H + L) Cross-Adsorbed Secondary Antibody, Alexa Fluor™ 647	Invitrogen	Cat# A21447; RRID:AB_2535864
Cy3-AffiniPure Donkey Anti-Rabbit IgG (H + L) (min X Bov,Ck,Gt,GP,Sy Hms,Hrs,Hu,Ms,Rat,Shp Sr Prot)	Jackson	Cat# 711-165-152; RRID:AB_2307443
Calnexin antibody	Cell Signaling Technology	Cat# 2433S; RRID:AB_2243887
OXPHOS Human WB Antibody Cocktail	Abcam	Cat# ab110411; RRID:AB_2756818
Anti-rabbit-IgG, HRP-linked antibody	Cell Signaling Technology	Cat# 7074; RRID:AB_2099233
Anti-mouse-IgG, HRP-linked antibody	Cell Signaling Technology	Cat# 7076; RRID:AB_330924

**Chemicals, peptides, and recombinant proteins**		

SB-612111	Merck	Cat# SML0571
Recombinant N/OFQ	Bio-Techne, Tocris	Cat# 0910, Batch no.: 018A
LPS	Sigma	Cat# L2630
Bovine Serum Albumin, Fatty Acid Free	Sigma	Cat# A8806
Huminsulin 100U/ml	Eli Lilly and Company	PZN: 02526396

**Experimental models: Cell lines**		

THP-1	ATCC	TIB-202, RRID:CVCL_0006
ES-Bruce 4	Dr. Frank Koentgen	N/A

**Experimental models: Organisms/strains**		

C57BL6/NTac	Taconic	N/A
B6.129P2(C)-Cd19^tm1(cre)Cgn/J^	Charles River	Rickert et al.^45^
Pnoc^tm2a(EUCOMM)Hmgu^	This paper	N/A

**Other**		

Maintenance Diet (NCD)	Ssniff Spezialdiäten	V1534-000
High-fat diet, 60 kJ% fat (HFD)	Ssniff Spezialdiäten	E15742-34

**Oligonucleotides**		

Mm-Hprt	Thermo Fisher Scientific	Mm03024075_m1
Mm-Tbp	Thermo Fisher Scientific	Mm01277042_m1
Mm-Pnoc (3–4)	Thermo Fisher Scientific	Mm00803087_m1
Mm-Adgre1	Thermo Fisher Scientific	Mm00802529_m1
Mm-Arg1	Thermo Fisher Scientific	Mm00475988_m1
Mm-Nos2	Thermo Fisher Scientific	Mm00440502_m1

**Software and algorithms**		

ImageJ Software	NIH	RRID:SCR_003070
Adiposoft plugin	NIH	https://doi.org/10.1194/jlr.D023788
Rstudio version 4.4.1	Posit	www.R-project.org; RRID:SCR_001905

**Deposited data**		

Mouse epididymal white adipose tissue RNA-Seq data	This paper	GSE281815
Mouse liver tissue RNA-Seq data	This paper	GSE281911
THP-1 macrophage RNA-Seq data	This paper	GSE281913

### Experimental model and subject details

#### Sex as a biological variable

Male mice were selected for metabolic phenotyping because, under the HFD paradigm used in this study, female mice exhibit attenuated weight gain.^64^^,^^65^ While the findings may be relevant to both sexes, studies in female mice are needed to confirm their broader applicability.

#### Animal husbandry

All animal procedures were conducted in compliance with protocols approved by local government authorities (Landesdirektion Sachsen, TVV06/22, T08/21). Mice were housed in individually ventilated cages (IVCs) at 22°C–24°C using a 12-h light/dark cycle. Animals had access to water and food *ad libitum*. Food was only withdrawn if required for an experiment. Unless otherwise stated, all experiments were performed using male mice.

#### Experimental diets

Experimental mice had *ad libitum* access to either a normal chow diet (NCD; V1534-000; ssniff Spezialdiäten GmbH) containing 67kJ% carbohydrates, 24kJ% protein and 9kJ% fat or a high-fat diet (HFD, E15742-34,corresponds to D12492, ssniff Spezialdiäten GmbH) containing 20kJ% carbohydrates, 20kJ% protein and 60kJ% fat. Mice were transitioned to HFD feeding at 8 weeks of age.

#### Generation of Pnoc-floxed (Pnoc^tm2a(EUCOMM)Hmgu^) mice

The plasmid containing a gene trap insertion (KO first allele, reporter-tagged insertion with conditional potential) in the *Pnoc* gene was obtained from the European Conditional Mouse Mutagenesis program (plasmid PG00256_Z_3_C10, EUCOMM).^66^ The targeting vector was electroporated into Bruce4 embryonic stem (ES) cells, and correctly targeted clones were identified using long-range PCR. For 5′ screening, positive clones were identified using the primers 5PNOC5SCREEN (sequence: ATGAAAATCCTCTTTTGTGACGTTCTGCTGCTCAGCCTGCTCTCCA) and 3LACZSCREEN (CCACCTCATCAGAAGCAGGCCACCCAACTGACCTTGGGCAA), yielding an expected band size of 6 kb. For 3′ screening, the primers 5NEO3SCREENPNOC (GGATCTAAGCTCTAGATAAGTAATGATCATAATCAGCCATATC) and 3PNOC3SCREEN (CTGACTCCCGGCCTGCAGGTCTTGGAGTGGACACATGCTGTA) were used, producing a 4.2 kb amplicon. The correctly targeted ES cell clones were injected into donor goGermline blastocysts. This system ensures exclusive germline transmission by eliminating the contribution of non-ES cell-derived germ cells.^67^ Chimeric mice were subsequently bred, and all offspring carried the ES cell-derived genome. Mice heterozygous for the targeted allele were crossed with Rosa26-FLPe mice (Jackson Lab Stock#: 016226) to generate floxed Pnoc mice. Pnoc-floxed mice were maintained on a C57BL/6NTac background. Animals were genotyped using primers flanking the 3′ loxP site: 5TYPPNOCFL (ACCTAACGGATCGAGTGAGTTAT) and 3TYPPNOCFL (CTAATGCCAGTCCACAGTAGTGC). This PCR yields a 221 bp fragment corresponding to the wild-type allele and a 285 bp fragment corresponding to the floxed allele.

#### CD19-Cre mice (B6.129P2(C)-Cd19^tm1(cre)Cgn/J^)

CD19-Cre mice were purchased from Charles River (Jackson Lab Stock #: 006785). The CD19-Cre allele contains a Cre recombinase gene inserted into the first coding exon of the CD19 gene. Cre recombinase is expressed from the earliest stages of B-lymphocyte development and persists throughout B-cell differentiation.^45^ CD19-Cre mice were backcrossed onto a C57BL/6NTac background.

#### *Pnoc*ΔCD19 mice

*Pnoc*^fl/wt^ mice were crossed with CD19-Cre mice backcrossed on an C57BL/6NTac background. Heterozygous breeding schemes (*Pnoc*^fl/wt^::CD19-Cre^tg/wt^ x Pnoc^fl/wt^::CD19-Cre^wt/wt^) were used to generate *Pnoc*^fl/wt^::CD19-Cre^tg/wt^ and *Pnoc*^fl/wt^::CD19-Cre^wt/wt^ mice. These mice were intercrossed to produce *Pnoc*^fl/fl^::CD19-Cre^tg/wt^ and *Pnoc*^wt/wt^::CD19-Cre^tg/wt^ control littermates. To account for potential effects of the Cre transgene or *Cd19* haploinsufficiency, we used CD19-Cre-expressing littermate controls throughout the study.

### Method details

#### THP-1 cells

THP-1 cells were maintained in RPMI 1640 (5mM glucose, 2 mM glutamine, 10 mM HEPES, and 1 mM sodium pyruvate, Gibco) supplemented with 10% fetal bovine serum (FBS), 100 U/ml penicillin and 100 μg/mL streptomycin, at 37°C with 5% CO_2_. Monocytic THP-1 cells were differentiated into macrophages by incubating the cells with 100 nM phorbol 12-myristate 13-acetate (PMA, P8139, Sigma-Aldrich) for 48 h, followed by an additional 72-h incubation in RPMI medium. Upon completion of the differentiation process, macrophages were detached using Trypsin-EDTA (0.25%, phenol red, Gibco) with a 5-min incubation at 37°C in a 5% CO_2_ humidified atmosphere. Detached cells were collected, transferred, and seeded at the required densities for subsequent experimental treatments. Differentiated macrophages were treated with either 1 ng/mL or 10 ng/mL of recombinant N/OFQ (Tocris Bioscience, Cat# 0910, Batch# 018A) for 24 h under standard culture conditions. Cell authentication was carried out by Eurofins Genomics (Germany) to confirm the correct identification of the THP-1 cells used in this study. The cells were tested negative for mycoplasma. None of the cell lines utilized were listed in the ICLAC database of commonly misidentified cell lines.

#### Bone marrow-derived macrophages

Bone marrow-derived macrophages (BMDMs) were isolated from the bone marrow of 14- to 16-week-old control animals. Mice were euthanized by cervical dislocation, and femurs and tibias were aseptically harvested. The bone marrow was flushed from the bones using a sterile syringe with ice-cold PBS into a sterile 50 mL Falcon tube. The cell suspension was passed through a 70 μm cell strainer and centrifuged at 300 x g for 5 min at 4°C. The resulting pellet was resuspended in red blood cell lysis buffer (Invitrogen, 00-4333-57) and incubated on ice for 5 min. Lysis was terminated by adding complete RPMI medium. The cells were centrifuged again at 300 x g for 5 min, and the pellet was resuspended in complete RPMI supplemented with L929-conditioned medium as a source of macrophage colony-stimulating factor (M-CSF). Cells were seeded at a density of 0.5 x 10^6^ cells/ml in 100-mm cell culture petri dishes and incubated at 37°C in 5% CO_2_. After 48 h, 5 mL of fresh complete RPMI containing L929-conditioned medium was added to the cultures. On day 5, the medium was fully replaced with fresh RPMI and L929-conditioned medium. By day 7, adherent macrophages were harvested via trypsinization and re-seeded for subsequent assays.

#### L929 cell-conditioned medium

L929 fibroblasts were rapidly thawed and immediately transferred into a 50 mL Falcon tube containing 20 mL of pre-warmed complete RPMI medium, supplemented with 10% FBS and 1% penicillin-streptomycin. The cell suspension was centrifuged at 1500 rpm for 5 min, and the resulting pellet was resuspended in 5 mL of complete RPMI medium. Cells were then seeded into 60-mm cell culture dishes and incubated at 37°C with 5% CO_2_ until reaching approximately 70% confluency. Upon reaching confluency, cells were trypsinized, centrifuged, and transferred to 150-mm dishes, each containing 30 mL of complete RPMI medium. The cells were maintained under these conditions for an additional 10 days. At the end of this incubation period, the conditioned medium was collected, filtered through a 0.45 μm filter to remove any residual cells, and aliquoted for storage at −20°C.

#### Adipose tissue conditioned media

Epididymal adipose tissue was collected under sterile conditions and thoroughly rinsed with sterile PBS. Using sterile scissors and forceps, the tissue was then minced into small fragments (approximately 1–2 mm). Minced adipose tissue was cultured in RPMI medium supplemented with 1% penicillin-streptomycin, as described in established protocols.^68^ After an initial 1-2-h rest period, the medium was checked under a microscope to verify the integrity of the explants. The explants were subsequently incubated for 48 h at 37°C in a humidified incubator with 5% CO_2_. After the incubation, the conditioned medium was collected, filtered through a 0.45 μm filter to remove remaining tissue debris, and aliquoted for storage at −80°C.

#### Transwell migration assay

To assess the migratory capacity of differentiated THP-1 macrophages, a 24-well transwell migration assay was performed. Differentiated THP-1 cells were seeded into the upper chambers of FluoroBlok transwell inserts (8 μm pore size, 734-0371, Corning) at a density of 100,000 cells per well in serum-free RPMI 1640 medium. Lower chambers were filled with RPMI 1640 medium supplemented with either 1 ng/mL or 10 ng/mL N/OFQ. Cells were allowed to migrate for 24 h at 37°C in a humidified atmosphere with 5% CO_2_. After the migration period, non-migrated cells were gently removed from the upper side of the membrane using a cotton swab. Migrated cells on the lower side of the membrane were fixed with 4% paraformaldehyde (PFA) in PBS at pH 7.4 for 15 min at room temperature. Following fixation, the inserts were washed once with PBS and stained with DAPI (D9542, Sigma Aldrich). Stained inserts were then placed in 24-well plates with PBS and individual inserts were imaged using a Keyence microscope (BZ-X800, Keyence) at 4× magnification. The number of migrated cells was quantified using Fiji software (ImageJ, NIH) by analyzing DAPI-stained nuclei. Transwell migration of non-differentiated THP-1 cells was assessed using 24-well plates with 8 μm FluoroBlok inserts (Corning). Cells (1 x 10^5^) were suspended in serum-free RPMI and placed in the upper chamber, while the lower chamber contained RPMI supplemented with recombinant N/OFQ (1 ng/mL). After 24 h at 37°C migrated cells were collected from the lower chamber and counted using an automated cell counter (CellDrop BF, DeNovix).

#### Seahorse XF96 mito stress test

Differentiated THP-1 cells were seeded at a density of 50,000 cells per well in Seahorse XFe96 cell culture microplates 24 h before measurement to ensure attachment of the cells. After seeding, cells were treated according to experimental conditions. The Seahorse XFe96 sensor cartridge was hydrated and pre-calibrated by adding 200 μL of XF calibrant to each well, followed by overnight incubation in a CO_2_-free environment at 37°C. On the day of the assay, the assay medium was prepared by supplementing Seahorse XF Base Medium with 10 mM glucose, 2 mM L-glutamine, and 1 mM sodium pyruvate, adjusting the pH to 7.4. The macrophages were gently washed with this assay medium and incubated for 45–60 min in a CO_2_-free incubator at 37°C to equilibrate. For analysis of non-differentiated THP-1 cells, Seahorse XFe96 microplates were coated with Cell-Tak (Corning, 354240) according to the manufacturer’s instructions. THP-1 cells were resuspended in assay medium (XF Base Medium supplemented with 10mM glucose, 2 mM L-glutamine, and 1 mM sodium pyruvate, pH 7.4) and seeded at a density of 50,000 cells per well. Plates were then centrifuged at low speed and incubated for 20 min at 37°C in a CO_2_-free incubator to facilitate immobilization. Non-adherent cells were gently removed by washing, and plates were equilibrated for 45–60 min prior to measurement. The following compounds were injected into designated ports: Oligomycin (1.5 μM in Port A), FCCP (2 μM in Port B) and Rotenone and Antimycin A (1 μM each in Port C). Each injection step was followed by real-time oxygen consumption rate (OCR) and extracellular acidification rate (ECAR) measurements. After the assay, data were analyzed using Seahorse Wave software.

#### B cell isolation

B cells were isolated from single-cell suspensions of mouse spleens using the Pan B Cell Isolation Kit (Miltenyi Biotec). Spleens were first dissociated into single-cell suspensions via mechanical dissociation with the gentleMACS dissociator (Miltenyi Biotec). The resulting suspension was passed through a 30 μm filter to eliminate cell clumps and debris, and erythrocytes were removed by lysis with Red Blood Cell Lysis Solution (00-4333-57, Invitrogen) prior to magnetic labeling. The cell suspension was incubated at 4°C with a biotin-conjugated antibody cocktail targeting non-B cells for 5 min, followed by a 10-min incubation with anti-biotin MicroBeads. After labeling, the cell suspension was applied to an LD column in a MACS Separator (Miltenyi Biotec). Non-B cells labeled with MicroBeads were retained within the column, while unlabeled B cells passed through and were collected for further analysis.

#### Analysis of gene expression

Total RNA was extracted from mouse tissues and THP-1 cells using the RNeasy Lipid Tissue Kit (Qiagen), with DNase treatment applied to all samples. RNA was then reverse transcribed using the High-Capacity cDNA Reverse Transcription Kit (Life Technologies). All subsequent qRT-PCR reactions were performed on a QuantStudio 7 Flex Real-Time PCR System (Applied Biosystems) using the Takyon Low ROX Probe 2X Master Mix (UF_NPMT-B0701, Eurogentec). For normalization, threshold cycles (Ct-values) of all replicate analyses were normalized to hypoxanthine guanine phosphoribosyl transferase (*Hprt*) or the TATA-binding protein (*Tbp*) housekeeping gene within each sample to obtain sample-specific ΔCt values (= Ct gene of interest - Ct Hprt). To compare the effect of various treatments with untreated controls, 2-ΔΔCt values were calculated to obtain fold expression levels, where ΔΔCt = (ΔCt treatment - ΔCt control). Probes were purchased from Applied Biosystems. The following TaqMan probes were used: *Pnoc* 3-4A (Assay ID: Mm00803087_m1), *Adgre1* (Assay ID: Mm00802529_m1), *Nos2* (Assay ID: Mm00440502_m1), *Arg1* (Assay ID: Mm00475988_m1). Calculations were performed using the comparative method (2^−ΔΔCt^).

#### RNA sequencing analysis

Total RNA was extracted, and RNA integrity was assessed using the Bioanalyzer 2100 system (Agilent Technologies). mRNA was purified from total RNA using poly-T oligo-attached magnetic beads. Fragmentation was followed by the synthesis of the first strand cDNA using random hexamer primers, and the second strand cDNA was synthesized using dUTP to ensure strand specificity. The resulting cDNA libraries underwent end repair, A-tailing, adapter ligation, size selection, amplification, and purification. Library quality was confirmed using Qubit for quantification, real-time PCR, and Bioanalyzer for size distribution. After validation, libraries were pooled based on concentration and sequencing requirements, and sequenced on an Illumina NovaSeq X Plus platform, generating paired-end reads of 75 base pairs. The raw sequencing data were first subjected to a quality control pipeline using fastp, which removed reads containing adapters, poly-N stretches, and low-quality sequences. Quality metrics, including Q20, Q30, and GC content, were calculated to ensure data integrity. The trimmed reads were aligned to the reference genome (GRCm39 for mouse data, GRCh38 for RNA sequencing of THP-1 cells) using HISAT2 v2.0.5, which generated a database of splice junctions from the gene model annotation file to improve alignment quality. Gene expression levels were quantified using featureCounts v1.5.0-p3, with results expressed in FPKM (Fragments Per Kilobase of transcript per Million mapped reads) to normalize for gene length and sequencing depth. For differential gene expression analysis, DESeq2 was employed for groups with biological replicates (minimum *n* = 3 per group). To control for multiple hypothesis testing, all *p*-values were adjusted using Benjamini–Hochberg false discovery rate (FDR) correction. Criteria for differential expression included log2 fold change >0.5 and adjusted *p*-value <0.05. Functional enrichment analysis was performed using the clusterProfiler R package for both Gene Ontology (GO) terms and KEGG pathways, identifying enriched biological processes and pathways. No post hoc filtering was applied to DEGs prior to gene set enrichment analysis.

#### RNA sequencing analysis of human visceral adipose tissue

The human data for this study were sourced from the cross-sectional cohort of the Leipzig Obesity Biobank (LOBB). This cohort comprises paired samples of abdominal subcutaneous and omental visceral adipose tissue from 1,480 participants. Among them, 31 individuals were classified as normal/overweight (52% women; average age: 55.8 ± 13.4 years; average BMI: 25.7 ± 2.7 kg/m^2^), while 1,449 were categorized as obese (71% women; average age: 46.9 ± 11.7 years; average BMI: 49.2 ± 8.3 kg/m^2^). Adipose tissue samples were collected during elective laparoscopic surgeries following established protocols. Body composition and metabolic parameters were assessed using standardized methods. Exclusion criteria included age under 18, a history of chronic substance or alcohol abuse, smoking within the year preceding surgery, acute inflammatory diseases, concurrent use of glitazones, end-stage malignancies, weight loss exceeding 3% within the three months prior to surgery, uncontrolled thyroid disorders, and Cushing syndrome. The study was approved by the Ethics Committee of the University of Leipzig (approval number: 159-12-21052012) and adhered to the principles outlined in the Declaration of Helsinki. All participants provided written informed consent before joining the study. We produced ribosomal RNA-depleted RNA sequencing data following the SMARTseq protocol. The libraries were sequenced as single-end reads on a Novaseq 6000 (Illumina, San Diego, CA, USA) at the Functional Genomics Center in Zurich, Switzerland. Preprocessing was carried out as previously described. In brief, adapter and quality-trimmed reads were aligned to the human reference genome (assembly GRCh38.p13, GENCODE release 32), and gene-level expression quantification was performed using Kallisto version 0.48. For samples with read counts over 20 million, we downsampled them to 20 million reads using the R package ezRun version 3.14.1 (https://github.com/uzh/ezRun, accessed April 27, 2023). Data normalization utilized a weighted trimmed mean (TMM) of log expression ratios, with adjustments made for age, transcript integrity numbers, and sex.

#### Re-analysis of scRNA-seq data of human hepatic immune cells

Single-cell RNA sequencing (scRNA-seq) data from healthy human samples were obtained from the Gene Expression Omnibus (GEO) database under accession number GSE136103.^69^ Eleven samples comprising CD45^+^ and CD45^−^ immune cell fractions from five healthy donors were selected for downstream analysis. Raw gene expression matrices in 10x Genomics format (including matrix, gene, and barcode files) were individually imported and used to generate separate Seurat objects. Each object was annotated with a unique sample identifier corresponding to its original metadata label. All samples were merged into a single Seurat object for joint analysis. Data were log-normalized, the top 2,000 most variable features were identified, and gene expression values were scaled. Dimensionality reduction was performed using principal component analysis (PCA), followed by Uniform Manifold Approximation and Projection (UMAP).

#### Western blotting

Protein samples were isolated using RIPA buffer (89901, Thermo Fisher) supplemented with protease and phosphatase inhibitors (5871S and 5872, Cell Signaling). Protein concentrations were determined using the Pierce BCA Protein Assay Kit (23227, Thermo Fisher). 25 μg protein were loaded onto 10–12% SDS-PAGE gels and separated by electrophoresis at 100–120 V. After electrophoresis, proteins were transferred to nitrocellulose membranes (0.2 μm, 10600053, Merck) using a wet transfer system overnight at 12V in ice-cold transfer buffer (25 mM Tris, 190 mM glycine, 20% methanol). Following transfer, membranes were stained with Ponceau Red solution to verify that transfer was correct. Followed by a blocking step in 3% BSA (Tris-buffered saline with 0.1% Tween 20, TBS-T) for 1 h at room temperature to prevent non-specific binding. Membranes were then incubated overnight at 4°C with primary antibodies diluted in 3% BSA in TBS-T. The following antibodies were used: Total OXPHOS Rodent WB Antibody Cocktail (1:500, Abcam ab110413). After three washes in TBS-T (5 min each), membranes were incubated with horseradish peroxidase (HRP)-conjugated secondary antibodies (1:5000; Cat no.7074; anti-mouse IgG; Cat no. 7076, Cell Signaling) for 1 h at room temperature. The membranes were washed again (3 times, 5 min each) in TBS-T and developed using an enhanced chemiluminescence (ECL) detection system with a G BOX IMAGER (Syngene). Calnexin was used as the loading control.

#### Histological analysis

Liver and adipose tissues were fixed overnight at 4°C in 4% PFA, dehydrated in graded ethanol, cleared in xylene, and embedded in paraffin wax according to standard protocols. Sections of 5 μm-thickness were cut using a microtome. Deparaffinization involved two 5-min immersions in xylene, followed by rehydration through graded ethanol concentrations (100% ethanol twice for 3 min each, then 95%, 80%, and 70% ethanol for 2 min each) and a 5-min rinse in distilled water. Sections were stained with hematoxylin for 5 min, rinsed in tap water for 5 min, differentiated briefly in acid alcohol (1% hydrochloric acid in 70% ethanol) to the desired intensity, and blued in tap water. A 2-min eosin Y counterstain was applied, followed by rapid dehydration through ascending ethanol concentrations (70%, 80%, 95%, and twice with 100% ethanol for 1–2 min each). Sections were cleared in xylene twice for 5 min each and mounted with Roti-Mount medium (Carl Roth). Slides were examined under bright-field illumination using a Keyence BZ-X800 microscope. Adipocyte size distribution was assessed using semi-automated morphometry. Specifically, three fields of view from three distinct sections (spaced at 200 μm intervals) per animal were analyzed. The automated cell quantification feature of Keyence BZ-X800 software was employed to identify and quantify adipocytes.

#### Immunohistochemistry

Immunohistochemistry was performed using the HRP/DAB detection IHC kit specific for mouse and rabbit antibodies (ab64264, Abcam), with an anti-MAC2 mouse monoclonal antibody (CL8942AP, Tebu-Bio). 5 μm sections were deparaffinized, rehydrated, and washed in distilled water prior to antigen retrieval. This was achieved by heating the sections in citrate buffer in a steam bath for 30 min, followed by cooling at room temperature for 20 min. Endogenous peroxidase activity was blocked by incubating the sections in hydrogen peroxide block for 10 min, followed by three 5-min washes in PBS-T buffer. To prevent non-specific binding, sections were then incubated with protein block for 10 min at room temperature, followed by a single wash in PBS-T. MAC2 polyclonal antibody (1:1000) was diluted in protein block buffer and applied to the sections overnight at 4°C in a humidified chamber. The following day, sections were washed four times with PBS-T, each wash lasting 10 min, and then incubated with a biotinylated goat anti-polyvalent secondary antibody for 10 min at room temperature, followed by four additional 10-min washes with PBS-T. Detection was performed using a DAB (3,3′-diaminobenzidine) substrate, with incubation timed to reach the desired staining intensity. Sections were counterstained with hematoxylin, dehydrated through a graded ethanol series, cleared in xylene, and mounted with Roti-Mount (Carl Roth). Stained slides were visualized using a Keyence BZ-X800 microscope, with MAC-2 positive cells counted per total adipocytes across four different high-power fields (10× magnification). For CD86 staining, liver sections were processed using the same immunohistochemistry protocol described above. Following antigen retrieval and blocking steps, sections were incubated overnight at 4°C with a rabbit monoclonal anti-CD86 antibody (1:500, Cell Signaling Technology, 19589T) diluted in protein block buffer. Detection was performed using the HRP/DAB system, and sections were counterstained with hematoxylin, dehydrated, cleared, and mounted as described. Representative images were acquired using a Keyence BZ-X800 microscope.

Triple immunofluorescence staining was used detect CD163, Mac2, and Perilipin. Slides were first deparaffinized in xylene (2 × 5 min), followed by rehydration through a descending ethanol series (100%, 95%, 70%; 3 min each). Antigen retrieval was performed using a citrate buffer (pH 6.0) in a pressure-based antigen retriever (Retriever 2100, Prestige Medical, Model No. 210045, 1500 W) for 20 min, followed by a 60-min cooldown at room temperature. After rinsing in distilled water, sections were permeabilized and washed in 0.3% Triton X-100 in PBS (5 × 5 min). Non-specific binding was blocked by incubating the sections for 1 h in 1% BSA (Roth, Cat. No. 3737.2) prepared in 0.3% PBS-Triton. Primary antibodies were diluted in 1% BSA in 0.3% PBS-Triton and applied overnight at 4°C: rat anti-Mac2 (1:1000, Cedarlane CL8942AP), rabbit anti-CD163 (1:100, Abcam ab182422), and goat anti-Perilipin A (1:200, Abcam ab61682). The following day, sections were washed again in 0.3% PBS-Triton (5 × 5 min; final two washes in cuvettes), followed by a 1-h incubation at room temperature with appropriate secondary antibodies (diluted 1:200 in 1% BSA in 0.3% PBS-Triton): donkey anti-rat Alexa Fluor 488 (Invitrogen SA5-10026), donkey anti-rabbit Cy3 (Jackson ImmunoResearch 711-165-152), and donkey anti-goat Alexa Fluor 647 (Invitrogen A21447). After secondary incubation, slides were washed (4 × 5 min in 0.3% PBS-Triton, all in cuvettes), stained with DAPI (1:10,000 dilution from a 25 mg/mL stock solution; SERVA 18860) for 10 min, and subsequently incubated with TrueBlack Lipofuscin Autofluorescence Quencher (1:20 dilution in ethanol; Biotium Cat. No. 23007) for 15 s. Finally, sections were washed twice in PBS and twice in distilled water (5 min each, in cuvettes), then mounted using DAKO Immunofluorescence Mounting Medium. Images were captured using a confocal microscope (LSM980, Zeiss, Oberkochen, Germany) equipped with 5x/0.25 and 10x/0.30 dry objectives. Laser intensities for the individual channels were kept constant throughout the imaging process.

#### Analysis of body composition

Body weights were measured weekly. Body composition was analyzed using Time-Domain Nuclear Magnetic Resonance (TD-NMR) on a Bruker Minispec LF50 system, with data acquisition and processing conducted automatically by the Minispec software.

#### Glucose tolerance tests

Glucose-tolerance tests (GTTs) were performed in mice that had been fasted for 16 h. Blood glucose concentrations were measured from whole venous blood using a handheld glucose monitor (Contour Ascensia, Bayer HealthCare, Germany). After determining the basal blood glucose concentrations, mice received an intraperitoneal injection of 20% glucose (10 mL per kg body weight; Braun). Blood glucose levels were subsequently measured at 15, 30, 60, and 120 min post-injection.

#### Insulin tolerance tests

For insulin tolerance tests (ITTs), animals were transferred to a new cage and food was removed. Basal blood glucose concentrations were measured before the mice received an intraperitoneal injection of 0.75 U/kg BW of human insulin (Huminsulin, Lilly). Blood glucose levels were then measured at 15, 30, and 60 min post-injection.

#### Indirect calorimetry

Indirect calorimetry was conducted using a PhenoMaster metabolic cage system (TSE Systems) to measure energy expenditure, respiratory exchange ratio, locomotor activity and food intake in individually housed mice. To ensure accurate baseline measurements, mice were acclimated to the chambers for 24 h prior to data collection, allowing them to adapt to the pellet and liquid dispensers and environmental conditions of the system. The climate-controlled chamber was set to 21°C with 50% humidity and a 12-h light-dark cycle. During the data acquisition phase, mice were provided *ad libitum* access to food and water, and metabolic parameters were recorded continuously over a 36-h period. Data points were collected at 5-min intervals.

#### Liver triglycerides

Triglyceride levels in liver samples were quantified using a Triglyceride Assay Kit (Abcam, Cat# ab65336). Frozen liver tissue (100 mg) was weighed and resuspended in 1 mL of 5% NP-40 in ddH_2_O. The tissue was homogenized using a Dounce homogenizer. Samples were heated at 95°C for 3 min, and this process was repeated to fully solubilize all triglycerides. The homogenates were centrifuged at maximum speed for 2 min to remove insoluble material. Supernatants were diluted 1:10 in ddH_2_O before proceeding with the assay. For the assay, standards and further diluted samples (1:25 dilution) were added to a 96-well microplate. To each sample and standard well, 2 μL of cholesterol esterase/lipase solution was added. For background correction, a separate set of wells was prepared for each sample without the addition of cholesterol esterase/lipase. The plate was incubated with constant agitation at room temperature for 20 min. Following the initial incubation, 50 μL of Enzyme Reaction Mix was added to each well. The Enzyme Reaction Mix consisted of 46 μL of Triglyceride Assay Buffer, 2 μL of OxiRed Probe, and 2 μL of Enzyme Mix VI (Triglyceride Enzyme Mix). The plate was then incubated for an additional 60 min, protected from light. Absorbance was measured at 570 nm using a Tecan Infinite M Plex microplate reader (Tecan, Cat# 30190085). For data analysis, the absorbance values from the background wells were subtracted from the readings of the total triglyceride wells to account for any endogenous glycerol or other interfering substances. Sample triglyceride concentrations were calculated by interpolating the corrected absorbance values from the standard curve.

#### Serum collection and analysis

Blood was collected via cardiac puncture, allowed to clot for 30 min at room temperature, and centrifuged at 3,000 x g for 15 min at 4°C. Serum was aliquoted to prevent multiple freeze-thaw cycles and stored at −80°C until analysis. On the day of the assay, samples were thawed on ice, analyzed in duplicate, and diluted according to the protocol specified for each assay. Serum insulin levels were determined using the Ultrasensitive Mouse Insulin ELISA Kit (Crystal Chem, Catalog #90080) according to the manufacturer’s instructions. Briefly, 5 μL of each serum sample and insulin standards were added to a 96-well plate pre-coated with anti-insulin antibodies, along with 95 μL of Assay Buffer. Subsequently, 100 μL of enzyme conjugate was added to each well, and the plate was incubated at 4°C for 2 h. After incubation, the wells were washed five times with wash buffer to remove unbound components. Next, 100 μL of conjugate solution was added to each well, and the plate was incubated at room temperature for 30 min, followed by seven additional washes. Afterward, 100 μL of substrate solution was added to each well, and the plate was incubated in the dark at room temperature for 40 min for low/wide range assays (or 10 min for high range assays). The reaction was terminated by adding 100 μL of stop solution to each well, and absorbance was immediately measured at 450/630 nm using a Flexstation 3 microplate reader (Molecular Devices). Serum leptin levels were measured using a Mouse Leptin ELISA Kit (Crystal Chem, Catalog #90030). Samples were diluted 1:20. Each well of a 96-well plate, pre-coated with anti-leptin antibodies, received 5 μL of serum sample and leptin standards, along with 95 μL of Assay Buffer. The plate was incubated overnight at 4°C. After incubation, the wells were washed five times, and 100 μL of enzyme conjugate was added, followed by a 4-h incubation at 4°C. After a further seven washes, 100 μL of substrate solution was added to each well, and the plate was incubated in the dark for 30 min at room temperature. The reaction was stopped by adding 100 μL of stop solution, and absorbance was measured immediately at 450/630 nm using a Flexstation 3 microplate reader (Molecular Devices). Serum N/OFQ levels were assessed using the Mouse N/OFQ ELISA Kit (Elabscience, Catalog #E-EL-M0862), following the manufacturer’s protocol. Serum samples were thawed on ice and diluted 1:1 (v/v) with the provided sample diluent. 50 μL of diluted serum and N/OFQ standards were added to wells pre-coated with anti-N/OFQ antibodies, followed by the immediate addition of 50 μL of Biotinylated Detection Antibody. The plate was incubated for 45 min at 37°C. After incubation, the wells were washed three times with wash buffer, followed by the addition of 100 μL of HRP-conjugate and a 30-min incubation at 37°C. After washing five times, 90 μL of substrate reagent was added to each well, and the plate was incubated at 37°C for 15 min. The reaction was terminated by adding 50 μL of stop solution, and absorbance was measured at 450 nm using a Flexstation 3 microplate reader (Molecular Devices).

#### Targeted proteomics analysis (OLINK)

Serum samples from HFD-fed mice were analyzed using the OLINK Mouse Target 48 cytokine panel. This method employs 43 pairs of protein-specific antibodies, each conjugated to partially complementary oligonucleotides (PEA probes). One microliter of serum was dispensed into each well of a 96-well plate, to which the antibody-oligonucleotide conjugates were added. Upon dual binding of the target protein by the corresponding antibody pair, the oligonucleotides were brought into close proximity, enabling hybridization. The hybridized oligonucleotides served as a template for DNA polymerase-mediated extension, generating a unique reporter DNA sequence specific to each target protein. These reporter amplicons were subsequently quantified by high-throughput microfluidic real-time PCR, allowing for multiplexed protein detection.

### Quantification and statistical analysis

All data are presented as mean ±standard error of the mean (SEM), unless otherwise specified. All figures and statistical analyses were generated using R version 4.4.1 (www.R-project.org) and GraphPad Prism 10 software. A *p*-value of <0.05 was considered statistically significant.
