# Supplementary material for: B cell-derived nociceptin/orphanin FQ contributes to impaired glucose tolerance and insulin resistance in obesity
Source: iScience. 2025 Jun 4;28(7):112819. doi: 10.1016/j.isci.2025.112819 (PMC12256348; doi:10.1016/j.isci.2025.112819)
Supplement: Document S1. Figures S1–S6 [file mmc1.pdf]

iScience, Volume *28*

Supplemental information

B cell-derived nociceptin/orphanin FQ contributes to impaired glucose tolerance and insulin resistance in obesity

Stephanie C. Puente-Ruiz, Leona Ide, Julia Schuller, Adel Ben-Kraiem, Anne Hoffmann, Adhideb Ghosh, Falko Noé, Christian Wolfrum, Kerstin Krause, Martin Gericke, Nora Klöting, Jens C. Brüning, F. Thomas Wunderlich, Matthias Blüher, and Alexander Jais

Supplementary Figures

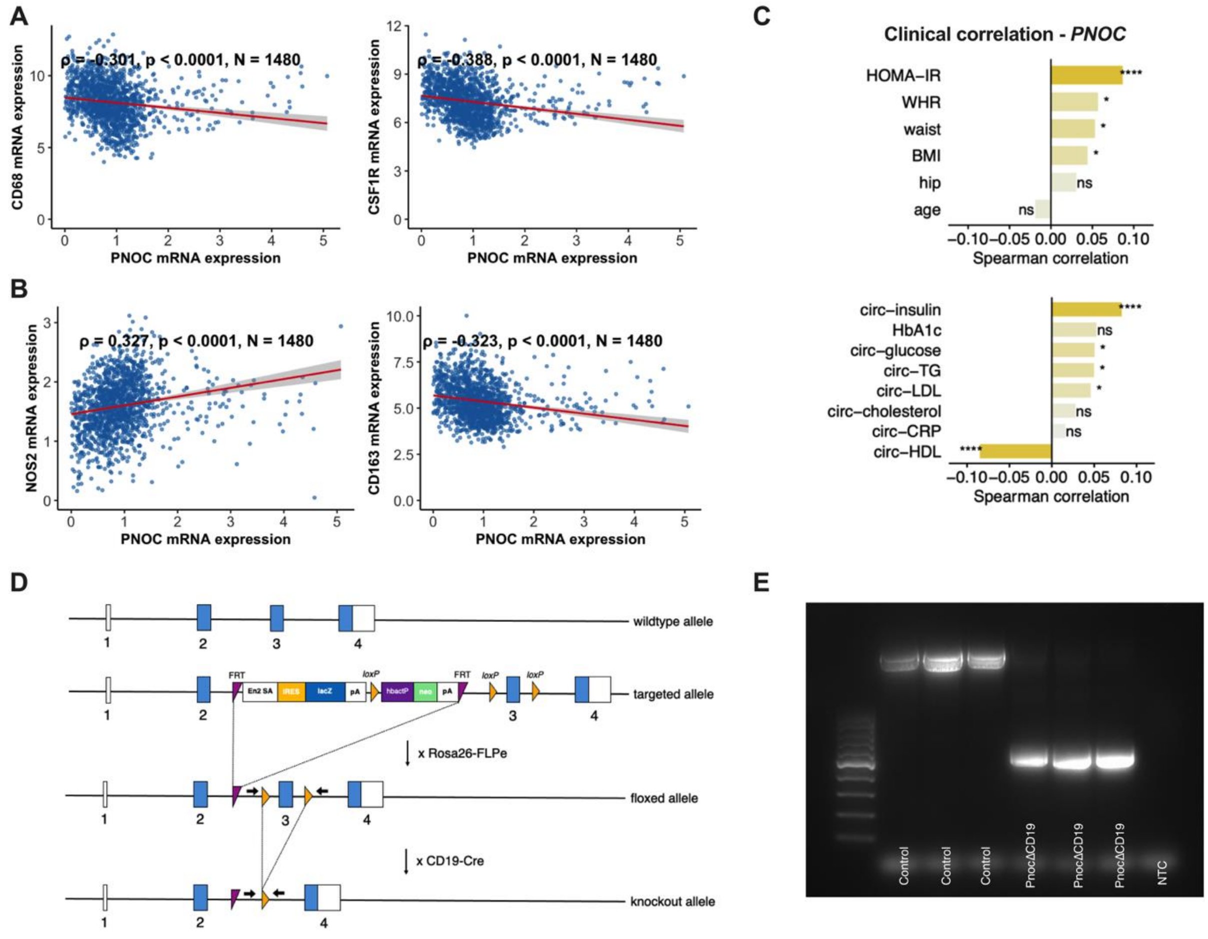

Supplementary Figure 1, related to Figure 1: Correlation of *PNOC* expression with B cell markers in human visceral adipose tissue

(A) Scatter plot showing the correlation between the expression levels of *CD68* and *PNOC*, as well as *CSFR1* and *PNOC*, in human visceral adipose tissue samples (n = 1,480, ρ = -0.301, p < 0.0001 for CD68; n = 1,480, ρ = -0.388, p < 0.0001 for CSFR1). **(B)** Scatter plot showing the correlation between the expression levels of *NOS2* and *PNOC*, as well as *CD163* and *PNOC*, in human visceral adipose tissue samples. (n = 1,480, ρ = 0.327, p < 0.0001 for *NOS2;* n = 1,480, ρ = -0.323, p < 0.0001 for *CD163*). **(C)** Clinical correlation of *PNOC* gene expression in human adipose tissue, as presented on the Adipose Tissue Knowledge Portal (adiposetissue.org)(44). **(D)** From top to bottom, the diagram shows the the wild-type (*Pnoc*) gene locus to the targeted, conditional (floxed), and knockout alleles. In the targeted allele, exon 3 of *Pnoc* is

flanked by loxP sites. Arrows indicate primer used to detect recombination of the knockout allele. **(E)** PCR analysis of knockout allele after Cre recombination.

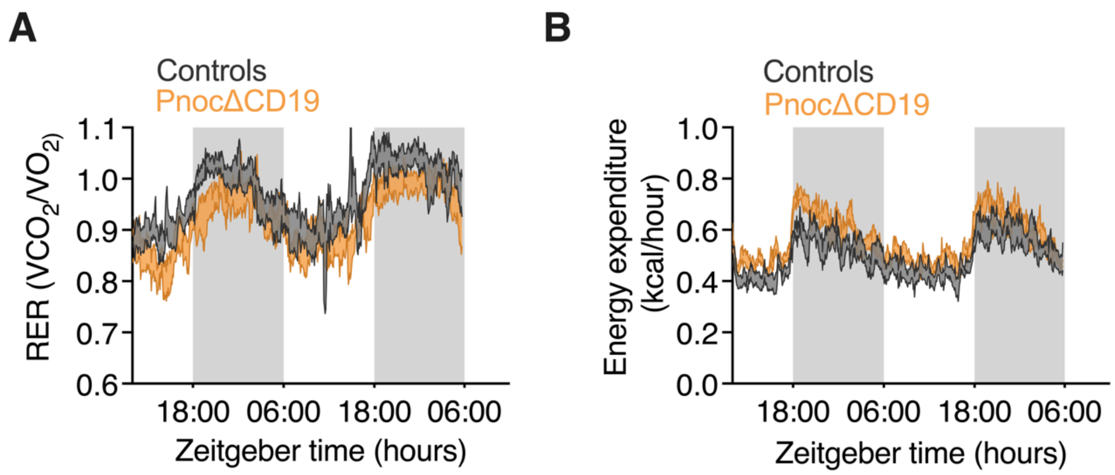

Supplementary Figure 2, related to Figure 2: Metabolic profiling of B cell-specific *Pnoc* knockout mice reveals mildly enhanced insulin sensitivity without changes in glucose tolerance

(A) Respiratory exchange ratio (RER) and **(B)** energy expenditure (EE) during two dark and two light cycles in 24-week-old control (n = 11) and *Pnoc*ΔCD19 mice (n = 11).

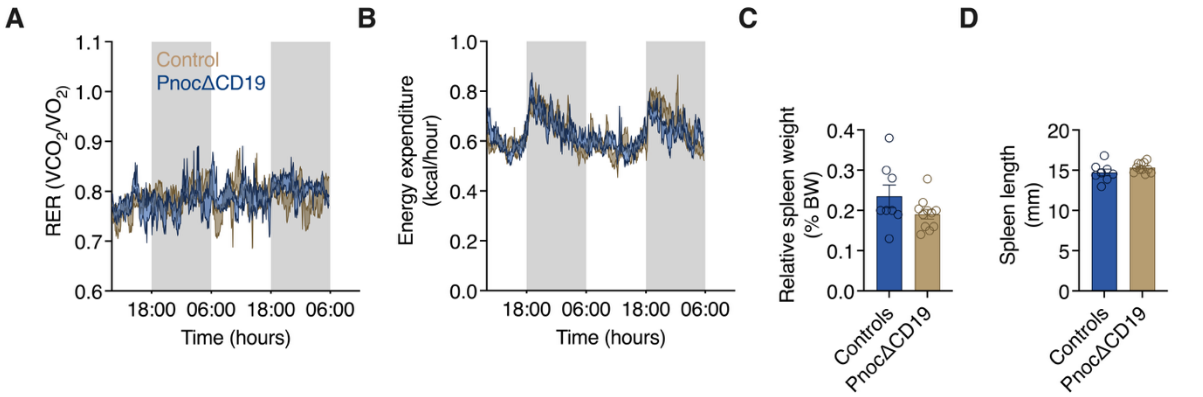

Supplementary Figure 3, related to Figure 3: B cell-specific *Pnoc* deletion enhances glucose tolerance and insulin sensitivity during high-fat diet feeding

(A) Respiratory exchange ratio (RER) and **(B)** energy expenditure (EE) in 16-week HFD-fed control (n = 7) and *Pnoc*ΔCD19 mice (n = 11) during two dark and two light cycles. **(C)** Spleen weight and **(D)** spleen of 16-week HFD-fed control (n = 8) and *Pnoc*ΔCD19 (n = 11) mice. Data are presented as mean ± SEM. Statistical analyses were performed using two-tailed Student's t-test. Significance levels are indicated as *p<0.05, **p<0.01, ***p<0.001.

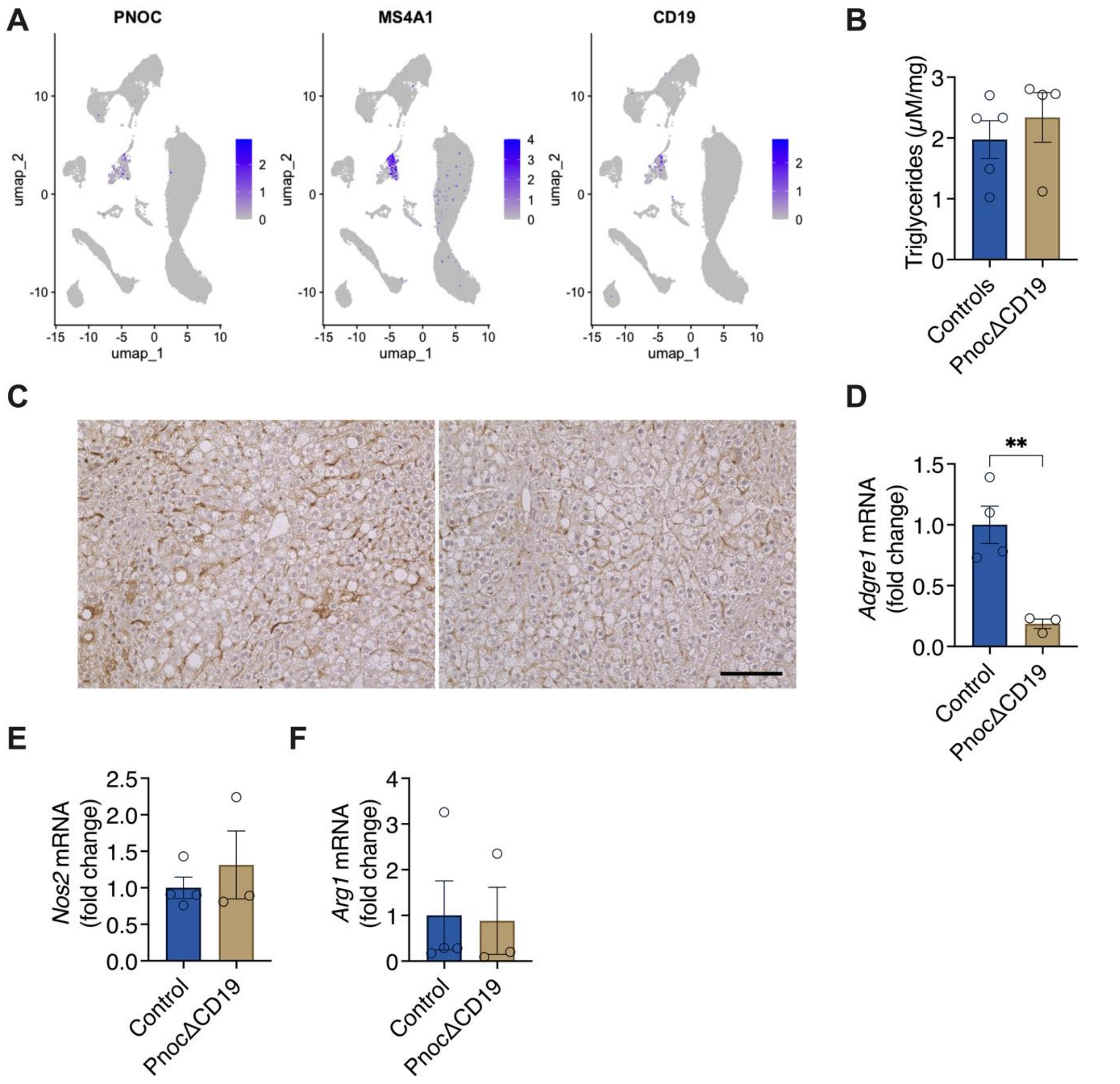

Supplementary Figure 4, related to Figure 4: B cell-specific *Pnoc* deletion alters immune cell recruitment in the liver under high-fat diet conditions

(A) UMAPs showing the expression of *PNOC* and B cell marker genes *CD19* and *MS4A1* in human hepatic B cells. **(B)** Hepatic triglyceride concentration from control (n = 5) and PnocΔCD19 (n = 4) mice fed an HFD. **(C)** Representative CD86 immunostaining of liver sections from HFD-fed control and PnocΔCD19 mice. **(D)** qPCR analysis of the pan-macrophage marker *Adgre1* (F4/80, Emr1) in liver samples from control (n = 4) and *Pnoc*ΔCD19 (n = 3) mice fed an HFD. **(E)** qPCR analysis of *Nos2* expression in liver samples from control (n = 4) and *Pnoc*ΔCD19 (n = 3) mice

fed an HFD. **(F)** qPCR analysis of *Arg1* expression in liver samples from control (n = 4) and *Pnoc*ΔCD19 (n = 3) mice fed an HFD. Data are presented as mean ± SEM. Statistical analyses were performed using two-tailed Student's t-test. Significance levels are indicated as *p<0.05, **p<0.01.

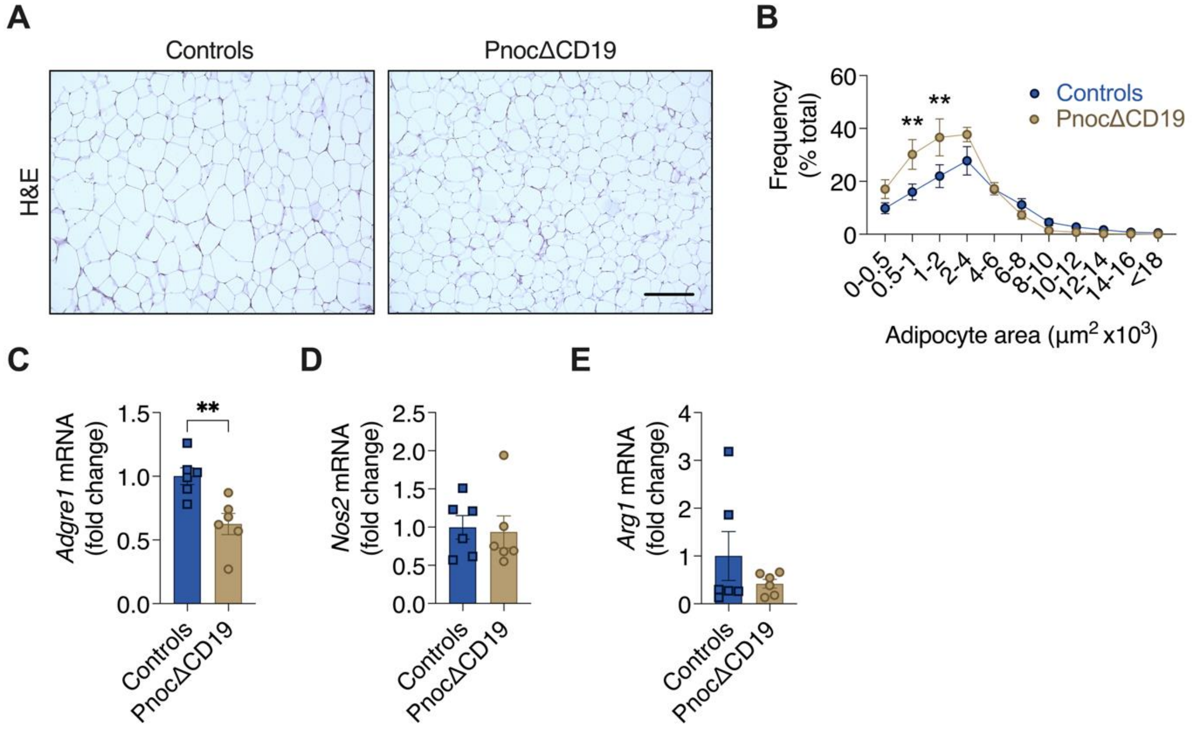

Supplementary Figure 5, Figure 5: B cell-specific *Pnoc* deletion alters macrophage recruitment and improves visceral adipose health under high-fat diet conditions

(A) Representative images of H&E staining of inguinal white adipose tissue (eWAT) from control and *Pnoc*ΔCD19 mice fed an HFD. Scale bar: 200 µm. **(B)** Quantification of adipocyte area in ingWAT from control (n = 5) and *Pnoc*ΔCD19 (n = 5) mice fed an HFD. **(C)** *Adgre1* (F4/80, Emr1) expression in ingWAT samples from control (n = 6) and *Pnoc*ΔCD19 (n = 6) mice fed an HFD. **(D)** *Nos2* expression in ingWAT samples from control (n = 6) and *Pnoc*ΔCD19 (n = 6) mice fed an HFD. **(E)** *Arg1* expression in ingWAT samples from control (n = 6) and *Pnoc*ΔCD19 (n = 6) mice fed an HFD.

Data are presented as mean ± SEM. Statistical analyses were performed using two-way ANOVA followed by Sidak’s multiple comparisons test (B) or two-tailed Student's t-test (C-F). Significance levels are indicated as *p<0.05, **p<0.01.

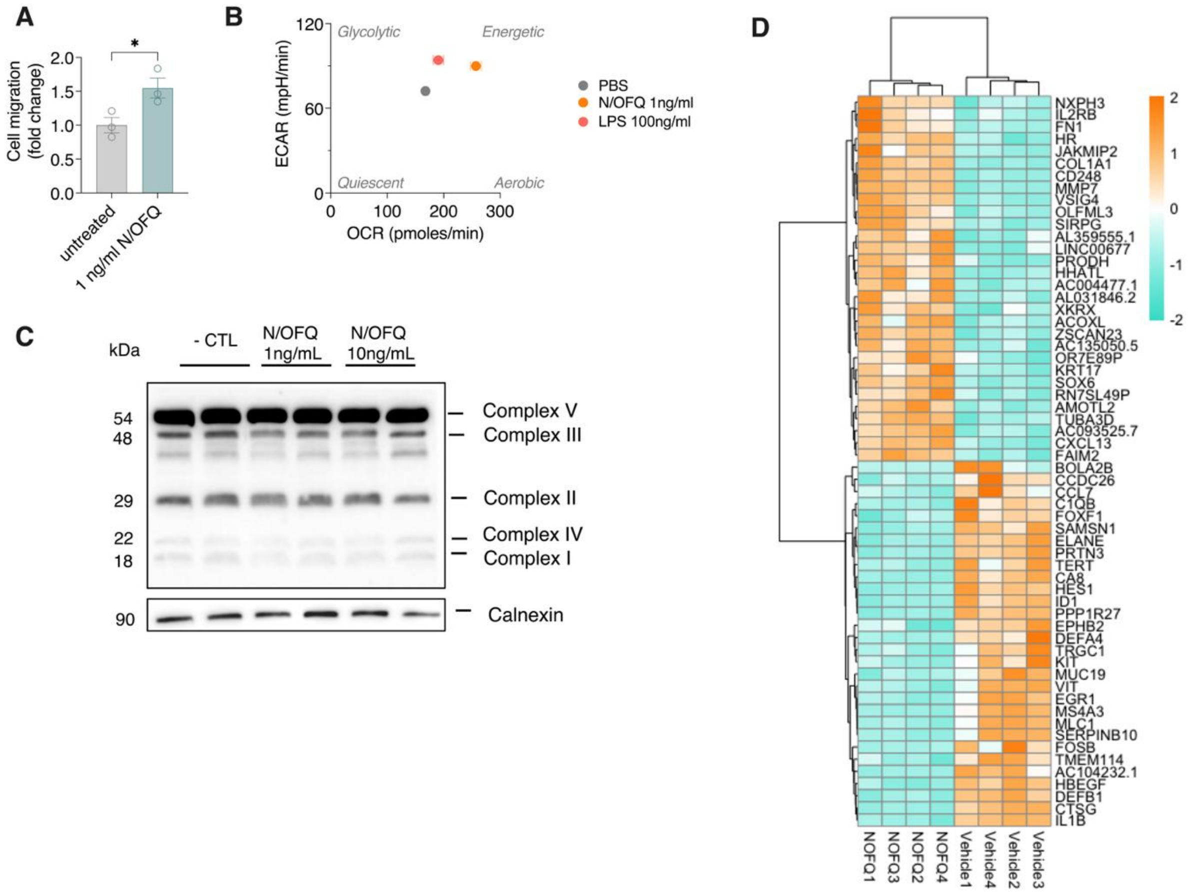

Supplementary Figure 6, related to Figure 7: N/OFQ enhances macrophage migration and bioenergetic metabolic phenotype through receptor-mediated chemotactic signaling

(A) Quantification of fold change in cell migration from transwell migration assays performed on undifferentiated monocytic THP-1 cells treated with 1 ng/ml recombinant N/OFQ for 24 hours (n = 3 independent experiments). **(B)** Seahorse analysis of THP-1 differentiated macrophages treated for 24 hours with 1 ng/ml N/OFQ or 100 ng/ml LPS (4 independent experiments, representative experiment is shown). Baseline oxygen consumption rate (OCR) as measure for respiration was plotted against the extracellular acidification rate (ECAR) as measure for glycolysis. **(C)** Protein extracts from THP-1 cells were analyzed with an antibody cocktail targeting components of the electron transport chain, with calnexin used as a loading control. **(D)** Heatmap illustrating the expression levels of differentially expressed genes in THP-1 differentiated macrophages treated for 24 hours with 10 ng/ml N/OFQ (n = 4). Data

are presented as mean ± SEM. Statistical analyses were performed using two-tailed Student's t-test (A). Significance levels are indicated as *$p<0.05$.
